# A SOX2-engineered epigenetic silencer factor represses the glioblastoma genetic program and restrains tumor development

**DOI:** 10.1126/sciadv.abn3986

**Published:** 2022-08-03

**Authors:** Valerio Benedetti, Federica Banfi, Mattia Zaghi, Raquel Moll-Diaz, Luca Massimino, Laura Argelich, Edoardo Bellini, Simone Bido, Sharon Muggeo, Gabriele Ordazzo, Giuseppina Mastrototaro, Matteo Moneta, Alessandro Sessa, Vania Broccoli

**Affiliations:** ^1^ Stem Cell and Neurogenesis Unit, Division of Neuroscience, IRCCS San Raffaele Scientific Institute, 20132 Milan, Italy.; ^2^ CNR Institute of Neuroscience, 20129 Milan, Italy.

## Abstract

Current therapies remain unsatisfactory in preventing the recurrence of glioblastoma multiforme (GBM), which leads to poor patient survival. By rational engineering of the transcription factor SOX2, a key promoter of GBM malignancy, together with the Kruppel-associated box and DNA methyltransferase3A/L catalytic domains, we generated a synthetic repressor named SOX2 epigenetic silencer (SES), which induces the transcriptional silencing of its original targets. By doing so, SES kills both glioma cell lines and patient-derived cancer stem cells in vitro and in vivo. SES expression, through local viral delivery in mouse xenografts, induces strong regression of human tumors and survival rescue. Conversely, SES is not harmful to neurons and glia, also thanks to a minimal promoter that restricts its expression in mitotically active cells, rarely present in the brain parenchyma. Collectively, SES produces a significant silencing of a large fraction of the SOX2 transcriptional network, achieving high levels of efficacy in repressing aggressive brain tumors.

## Teaser

The SOX2 epigenetic silencer (SES) is a synthetic GBM inhibitor factor that represses the SOX2 oncogenic transcriptional network.

## INTRODUCTION

Glioblastoma multiforme (GBM) is the most common and lethal brain cancer in adults, with one to five cases per 100,000 people per year and a median survival time of 12 to 15 months (*1*). This poor outcome is due to the combination of both the aggressiveness of the disease and the limited efficacy of current therapies that only marginally increase the overall survival (*2*, *3*). Patients usually undergo surgical resection of the primary tumor mass followed by adjuvant radio- and chemotherapies [temozolomide (TMZ)] that, however, fail to prevent tumor recurrence in virtually all cases (*2*–*4*). It has been proposed that even if surgery is radical as possible, the few remaining cancer cells in the healthy tissue with tumor-initiating potential are sufficient to regrow the tumor mass in the short term, leading to recurrence of the disease. In particular, cancer stem cells (CSCs), defined as cells able to self-renew and reform the tumor, remain quiescent or have very low proliferative activity and are capable of developing resistance to adjuvant treatments (*5*–*7*). Thus, there is an urgent medical need to achieve long-lasting remission after tumor resection and to develop an efficient strategy for targeting residual cancer cells and suppressing their tumor activity.

Many efforts have been focused on investigating the molecular program of CSCs to find and eventually inactivate genes fundamental for their survival and malignant properties. However, they often present different features and intrinsic heterogeneity as the characteristics of the tumor of origin (*7*–*11*). It has been proposed that glioblastoma heterogeneity, due to genetic, epigenetic, and microenvironmental influences on cellular programs, may be the basis of therapeutic failure (*12*). One aspect of GBM heterogeneity is reflected by the variable transcriptional makeup of the different GBM subtypes, e.g., classical, mesenchymal, and proneural, which are partially enriched for genetic events such as alterations in *PDGFRA* (proneural subtype) and *EGFR* (classical) (*13*, *14*). Single-cell transcriptomic studies have also revealed that these subtype programs can coexist in the same tumor either in different regions, at different times, or as a result of therapeutic regimens (*11*, *14*). A second layer of heterogeneity is the developmental status of GBM cells within the tumor. It has been demonstrated that, across different tumor samples and subtypes, GBM contains different proportions of malignant cells that exist in neural progenitor cell–like, oligodendrocyte progenitor cell–like, astrocyte cell–like, and mesenchymal-like states that are plastic enough to undergo transition from one to another (*11*). For instance, it has been reported that, following current clinical treatments, the remaining tumor cells transit into a mesenchymal phenotype, suggesting that an epithelial-to-mesenchymal (EMT)–like mechanism in GBM is associated with therapy resistance (*15*–*18*). Specific genetic drivers might favor certain transition probabilities and define the distribution of the different cell states either under physiological conditions or in response to treatments. This, on the one hand, explains why targeting a single gene in GBM often shows limited efficacy and, on the other hand, suggests that strategies modifying the entire molecular pathways may offer new avenues for GBM treatment.

CSCs have many features in common with neural stem cells in the healthy developing brain, with both cell types sharing transcription factors (TFs) that are fundamental for their vitality and proliferation. Thus, scientists carried out several attempts to restrain GBM development by silencing one or more of these TFs with different technologies. Among the oncogenic TFs, SOX2 is of particular importance because it is necessary for the CSCs of most gliomas; it is mostly silenced in differentiated cells (*19*) and operates as a super pioneer TF (*20*, *21*). From a therapeutic perspective, *SOX2* gene inactivation has been attempted with several technologies, including short hairpin RNA–, microRNA-, and transcription activator–like effector nuclease (TALEN)–based epigenetic repressors, but complete and long-term gene silencing has proven difficult to achieve (*21*). Moreover, cancer cells can easily rearrange their genetic program to cope with the silencing of a single gene and, thus, maintain unaltered tumorigenic potential (*14*). In principle, it would be more effective to silence the entire SOX2 transcriptional network rather than the single TF itself. Because SOX2 has thousands of putative direct target genes along the genome (*19*), cancer cells hardly overcome these changes. To this end, we designed and validated an engineered SOX2 factor, coined SOX2 epigenetic silencer (SES), which functions as an epigenetic repressor, to switch off the SOX2 downstream oncogenic gene network and, thus, inhibit CSC survival and proliferation. SES retained the original ability of SOX2 to recognize and bind to its own targets in the genome without the possibility of activating their transcription but rather permanently silencing them by inducing stable epigenetic modifications. Local viral gene delivery in the brain tissue granted efficient targeting of the cancer cells, enabling the expression of SES, which elicited strong tumor regression with no side effects. Thus, rational engineering of the oncogenic TF SOX2 guided the development of a synthetic epigenetic silencer with strong antitumor activity in brain cancer.

## RESULTS

### Generation of the SOX2 epigenetic silencer

**Fig. 1.**
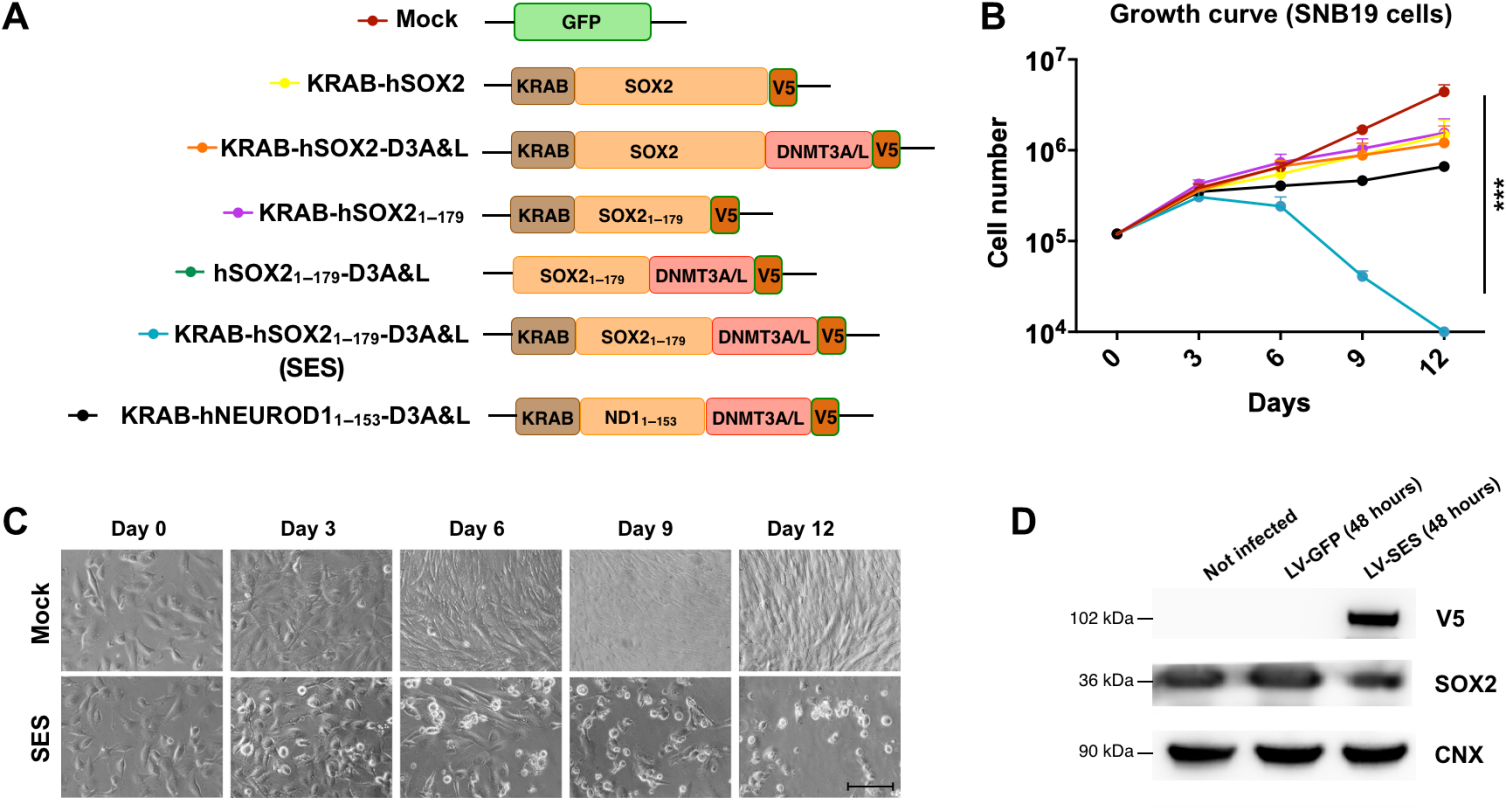
Generation and in vitro efficacy of the SOX2 epigenetic silencer. (**A**) Constructs were generated on the basis of human SOX2 TF and the epigenetic domain KRAB, DNMT3A (3A), and DNMT3L (3L), and V5 was added as tag. A factor based on human NEUROD1 TF and the epigenetic domain KRAB, DNMT3A (3A), DNMT3L (3L), and V5 were added as additional control. (**B**) Growth curve of SNB19 cells infected with the indicated constructs indicates that SES is able to kill the cells after 12 days in culture. ***P = 0.001; statistically compared with two-way analysis of variance (ANOVA). *n* = 3. (**C**) Microphotographs of the cells at the indicated time points from the infection with either mock-expressing (GFP, green fluorescent protein) or SES-expressing lentiviruses. (**D**) Western blot (WB) for V5, SOX2, and calnexin (CNX) (as loading control) in SNB19 cells either not infected or infected with lentivirus carrying GFP or SES. Scale bar, 100 μm (C).

We sought to build a set of SOX2 epigenetic repressors by fusing the Kruppel-associated box (KRAB) domain and/or the catalytic domain of the DNA methyltransferase DNMT3A together with its cofactor DNMT3L to the full-length SOX2 sequence or the SOX2 sequence lacking the C-terminal transcriptional activation domain (Fig. 1A). The KRAB domain (from the zinc finger protein ZNF10) recruits different epigenetic complexes able to both induce repressive chromatin modifications (e.g., H3K9me3) and remove active marks (e.g., H3K4ac), while the DNMT domains coordinate de novo DNA methylation, thus in combination repressing gene transcription (*22*, *23*). The sole presence of either KRAB or DNMTs fused with SOX2 (both full-length and without C-terminal domain) was not effective in blocking SNB19 glioma cells in vitro (Fig. 1, A and B); thus, we decided to combine them together. Computational modeling of the protein structures was developed using the I-TASSER and, on the basis of the crystal structure of the SOX2 high-mobility group (HMG), KRAB, and DNMT3A/L domains, was performed to guide to the best experimental option between three possible domain configurations (fig. S1A and table S1). The in silico prediction suggested that the factor, named SES onward, composed of the N-terminal and DNA binding regions of SOX2 plus the KRAB and DNMT domains at the 5′ and 3′ terminals, respectively, showed the best folding stability among the generated models (fig. S1A and table S1). Moreover, the assembly of the repressor domains within SES should not alter the DNA binding domain in the synthetic factor (fig. S1B) (*24*).

**Fig. 2.**
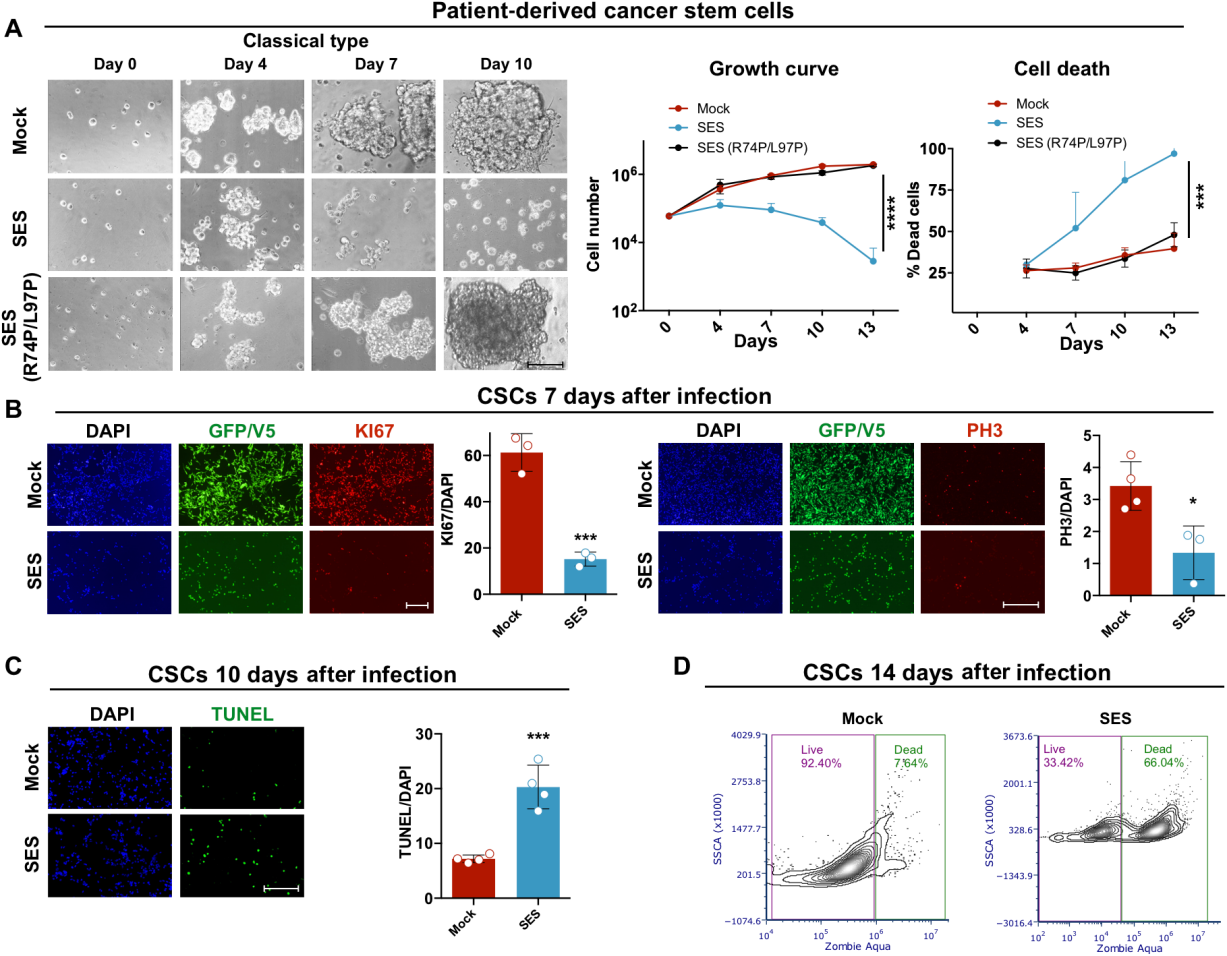
SES efficacy in patient-derived CSCs. (**A**) Microphotographs, growth curve, and percentage of dead cells (trypan blue automatic counting) of patient-derived CSCs of classical GBM subtype infected with either GFP (mock), SES, or SES (R74P/L97P). Growth curve: ****P < 0.0001; dead cells: ***P = 0.007; statistically compared with two-way ANOVA. *n* = 3. (**B**) Left: Immunocytochemistry for KI67 mitotic marker and for GFP and V5 tag, counterstained with 4′,6-diamidino-2-phenylindole (DAPI) in CSCs 7 days after GFP or SES viral infection. Quantification as percentage of KI67^+^ cells on the total number of DAPI nuclei (means ± SEM); ***P = 0.0008; statistically compared using unpaired *t* test. *n* = 3. Right: Immunocytochemistry for PH3 (marker for mitoses) and for GFP and V5 tag, counterstained with DAPI in CSCs 7 days after GFP or SES infection. Quantification as percentage of PH3^+^ cells on the total number of DAPI nuclei (means ± SEM); *P = 0.0183; statistically compared using unpaired *t* test. *n* = 3. (**C**) Terminal deoxynucleotidyl transferase–mediated deoxyuridine triphosphate nick end labeling (TUNEL) assay counterstained with DAPI in CSCs 10 days after GFP or SES infection. Quantification as percentage of KI67^+^ cells on the total number of DAPI nuclei (means ± SEM); ***P = 0.0006; statistically compared using unpaired *t* test. *n* = 3. (**D**) Fluorescence-activated cell sorting analysis using the Zombie Aqua dye staining for assessing cell death in both mock- and SES-infected CSCs after 14 days from the infection. Gating strategy was based on unstained cells. Scale bars, 100 μm (B left), 300 μm (A), and 200 μm (B right and C). SSCA, side scatter area.

SES rapidly killed SNB19 and U251 glioma cell lines in vitro (Fig. 1, B to D, and fig. S2, A and B). We found that SES expression reduced PH3^+^ proliferative cells (fig. S2C) and stimulated cell death, as assessed by flow cytometry with the dead cell–penetrant dye Zombie Aqua (fig. S2D). We extended this analysis to patient-derived GBM CSCs of classical subtype that better preserve primary tumor features. GBM CSCs displayed a strong proliferative loss and high cell death after SES lentiviral (LV) transduction (Fig. 2, A to D). In addition, clonogenic analysis showed that SES-treated CSCs exhibited a diminished self-renewal capacity with impairment in forming tumor spheres and in sustaining their growth (fig. S2E). We also confirmed the proliferative loss induced by SES in CSCs of the proneural subtype (fig. S2F). To determine SES specificity, we mutated two residues in the HMG-box domain (arginine in position 74 and leucine in position 97 replaced by two prolines) that were described to prevent SOX2 to bind the DNA (Fig. 1A) (*25*). Expression of SES (R74P/L97P) failed to arrest CSC growth, indicating that SES activity relies on its DNA binding activity. To further ascertain the specificity of this protein engineering, we substituted the DNA binding domain of SOX2 with that from NEUROD1 (NEUROD1_1–153_), a basic helix-loop-helix TF involved in neuronal differentiation (*26*, *27*), unrelated with GBM malignancy (Fig. 1A). The acute overexpression of KRAB-hNEUROD1_1–153_-DNMT3-3L did not noticeably alter the growth of SNB19 cells (Fig. 1B), supporting the specific molecular action of SES activity responsible for its antitumor effects.

Then, we sought to understand whether SES can be repressed over time, e.g., by long terminal repeat silencing. In the normal approach with LV, after 7 days from the infection, SES mRNA decreased while the green fluorescent protein (GFP) transcript increased (fig. S3A). Nevertheless, the presence of the episomal vector and the proliferative disadvantage of SES-expressing cells in vitro resulted in a stronger decrease of vector genome copy number, thereby compromising the subsequent analysis (fig. S3A). Thus, we cloned the synthetic factor in an LV with Flex configuration (*28*), which permits the expression of the transgene only after CRE recombinase–mediated flipping of the cassette (fig. S3, B and C). The puromycin resistance allowed the establishment of a CSC stable line carrying the vector integrated (SES off) without episomal contamination and proliferative bias. After CRE-mediated recombination, the SES expression was detectable in virtually all cells (as scored for V5 tag positivity) and was constant (viral mRNA levels), demonstrating that the transgene is not silenced over time (fig. S3, D and E).

**Fig. 3.**
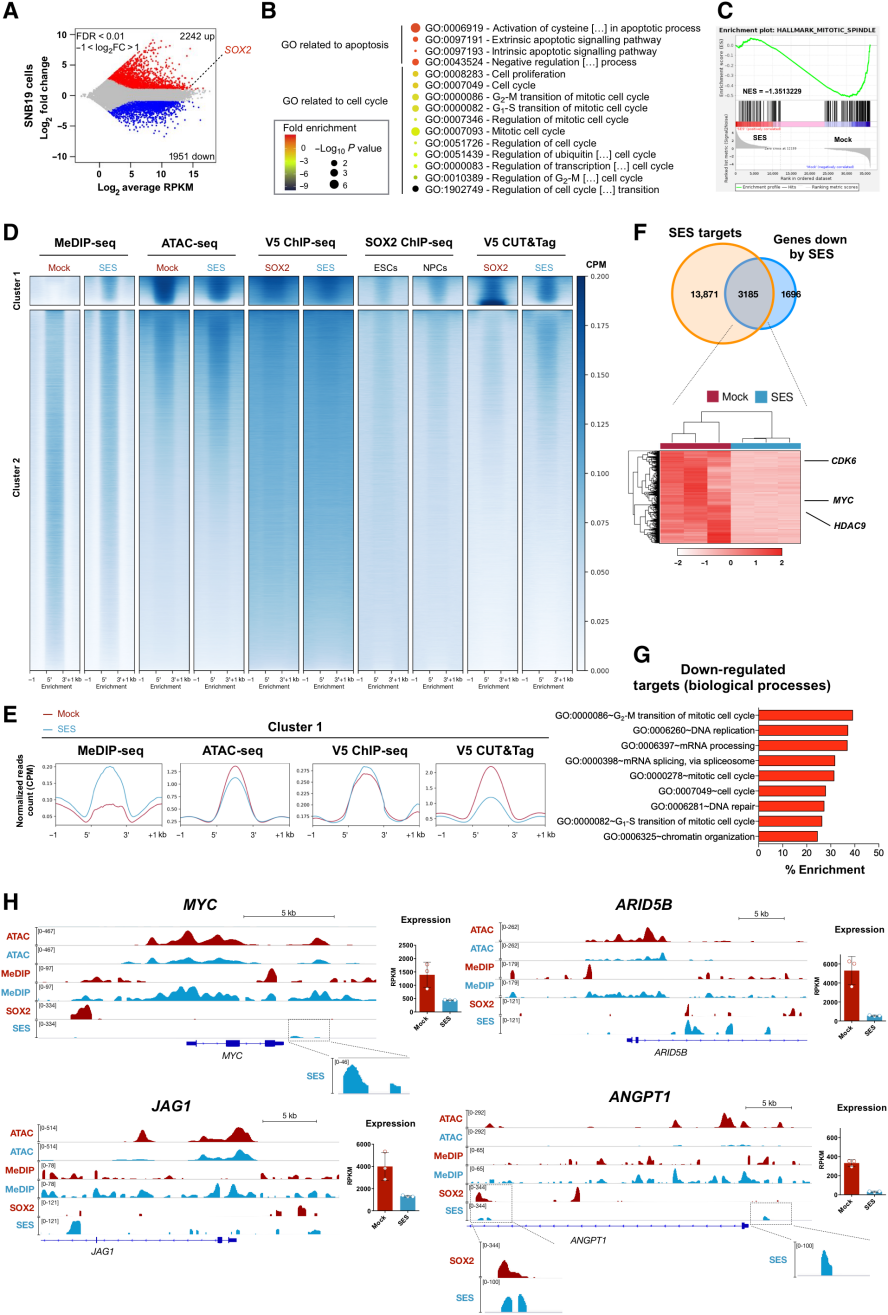
SES induces extensive transcriptomic and epigenomic changes. (**A**) SES causes massive gene deregulation in SNB19 cells 2 days after the infection, as assessed by RNA sequencing (RNA-seq). FDR, false discovery rate; FC, fold change; RPKM, reads per kilobase of transcript per million. (**B**) Gene Ontology (GO) analysis indicates that genes associated with apoptosis (up-regulation) and cell cycle regulation (down-regulation) are impaired by SES expression. (**C**) Enrichment plots from gene set enrichment analysis for Hallmark Mitotic spindle (see also table S2). NES, normalized enrichment score. (**D**) Heatmaps showing relative enrichment for MeDIP-seq, ATAC-seq, ChIP-seq (V5 tag for SOX2 and SES overexpression), and CUT&Tag (V5 tag for SOX2 and SES overexpression) in both control and SES conditions as well as data from publicly available ChiP-seq for endogenous SOX2 in ESCs and NPCs (GSE69479) in all the called peaks (*n* = 1,048,756 unique rows). Enrichments show as color scale in peak bodies of ±1 kb. (**E**) Density plots summarizing the mean of the signals for the indicate dataset in the regions belonging cluster 1 in (D). (**F**) Top: Venn diagram illustrating the overlap between genes with regions of cluster 1 in (D) (SES targets) and genes down-regulated (FDR < 0.1; FC < 0). Bottom: Heatmaps with genes both direct targets and down-regulated by SES (3185 rows). (**G**) GO analysis indicates that genes associated with chromatin organization, mRNA processing, and cell cycle regulation are enriched among genes that are both SES targets and down-regulated by its expression. (**H**) IGV (Integrative Genomics Viewer) snapshots of SES targets showing both ATAC-seq, MeDIP-seq, and V5–ChIP-seq tracks in both mock-infected (red tracks) and SES-infected (blue tracks) cells. Strong chromatin remodeling is observed nearby the SOX2/SES binding sites. Expression data (RPKM from RNA-seq experiments) are also shown on the right.

Next, we set out to assess global SES transcriptional output and its genome-wide occupancy. SES-treated glioma cells exhibited massive transcriptional changes with significant up-regulation of apoptosis-related genes and silencing of genes encoding proliferative and cancer-promoting factors (Fig. 3, A to C; fig. S4, A and B; and table S2). Then, we performed experiments to determine the genome-wide binding of both SOX2- and SES-overexpressing factors. Using chromatin immunoprecipitation sequencing (ChIP-seq), we showed that SES- and SOX2-bound genomic sites in cancer cells are similar to the endogenous SOX2 footprint in human neural precursors (NPCs) and, to a lesser extent, in embryonic stem cells (ESCs) (Fig. 3D). However, the quality of the signal was low, making peaks hard to be properly determined. Thus, we expanded our analysis using the cleavage under targets and tagmentation (CUT&Tag) assay (*29*), through which we succeeded to identify discrete peaks for both SOX2 and SES (Fig. 3D and table S3). With this technique, we revealed that about 25% of SES peaks are in common with SOX2 genomic sites in cancer cells (fig. S4C and table S3), while most of the remaining peaks (SES only) localized nearby SOX2 binding regions (fig. S4D). Both datasets contained enrichment for the TF binding sites of the SOX family (fig. S4E and table S4) and showed a general overlap between them and SOX2 ChIP-seq tracks (Fig. 3D). Given that SES includes the catalytic domains of the DNMT3A/L de novo DNA methyltransferases, we exploited methylated DNA immunoprecipitation sequencing (MeDIP-seq) to profile methylated DNA and assay for transposase-accessible chromatin using sequencing (ATAC-seq) to evaluate chromatin accessibility upon both mock (GFP) and SES treatment (Fig. 3D). At a genome-wide level, we noticed that regions with highly bound by SES and SOX2 (CUT&Tag peaks), and overlapping with the SOX2 fingerprint consensus, showed a strong increase in the methylation state with a concomitant decrease in chromatin accessibility level, suggesting an epigenetic reconfiguration on the basis of the SES transcriptional silencing (Fig. 3, D, cluster 1, and E, and table S3). SES binding was weak (albeit present) in the regions in which the SOX2 binding was stronger (fig. S4F, cluster 2, and table S3), possibly reflecting a competition between endogenous SOX2 and SES in treated cells. However, this antagonism did not prevent SES activity because the loss of chromatin accessibility was higher in these regions (fig. S4F, cluster 2) than in those SES binding prevailed (fig. S4F, cluster 1). Accordingly, most of the down-regulated genes were bound by SES (Fig. 3F and table S5) and exhibited higher gain of DNA methylation than genes that were not down-regulated (fig. S4G). Down-regulated SES targets comprise key genes for cell cycle execution, chromatin organization, and DNA replication, among which are *MYC*, *ARID5B*, *JAG1*, and *CDK6* (Fig. 3, F to H; fig. S4H; and table S5). Epigenetic rewiring was evident by the loss of chromatin accessibility and the increase in DNA methylation, which might span several kilobases around the binding site (Fig. 3H and fig. S4H). We observed that the methylation gained in SES-treated cells was sustained in a longer spatial window when a CpG island (CGI) is present within the region (fig. S4I). On the same line, long [>500 base pairs (bp)] MeDIP peaks were enriched for both CGI coverage (as a percentage of the linear genome of the peaks that falls in CGI) and CpG dinucleotide abundance, compared to short peaks (<500 bp) (fig. S4J). Genomic analyses were conducted 2 days postinfection (DPI); thus, the effects on both chromatin and gene transcription could not be achieved on certain targets. At 4 DPI, at least some of the target genes that were unchanged at the early time point were down-regulated afterward (fig. S4K). These data indicate that SES, by repressing, at least part, of the SOX2 genetic network, is capable of strongly inhibiting cancer cell proliferation and survival.

### SES antitumor activity in mouse xenograft models

**Fig. 4.**
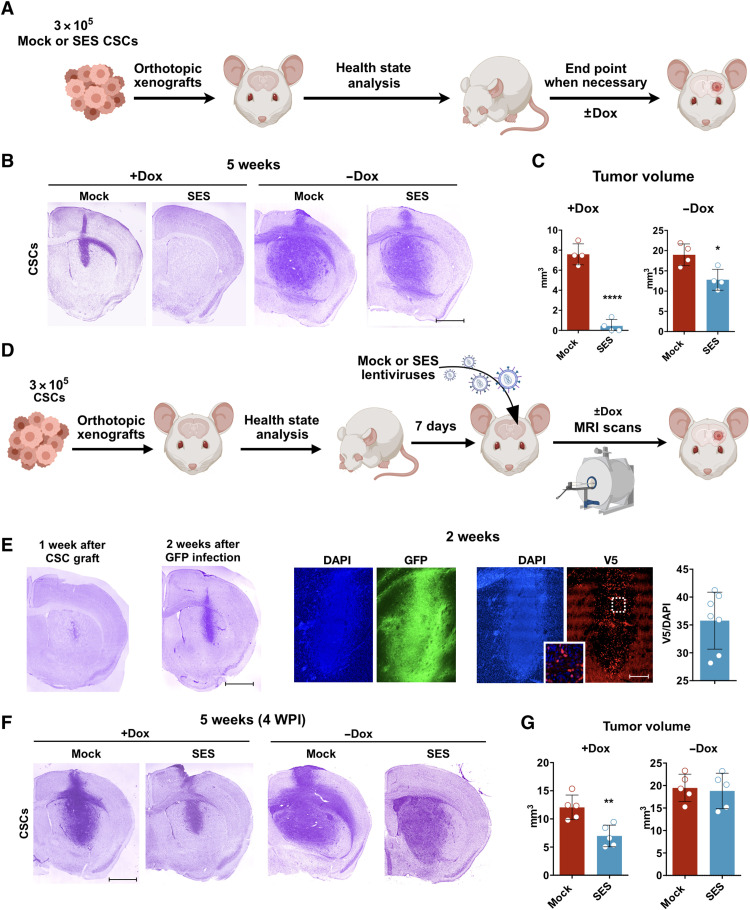
SES treatment inhibits orthotopic xenograft growth in immunodeficient mice. (**A**) Schematic representation of the orthotopic xenograft by brain injection (striatum) of 300,000 CSCs (classical type), preinfected with either mock (Tta and TetOn-GFP) or SES (rtTA and TetOn-SES) in NSG mice (±dox). (**B**) Nissl staining of representative section of the indicated models 5 weeks after the transplant. (**C**) Quantification of tumor volume (four animals per group, means ± SEM): +dox: ****P < 0.0001; −dox: *P = 0.0164; statistically compared with unpaired *t* test. *n* = 4 from four tumors. (**D**) The orthotopic xenograft is generated by injection of 300,000 undifferentiated CSCs (classic type) in the striatum of NSG mice; after 7 days, the animals are reoperated to inject lentivirus carrying either mock (rtTA and TetON-GFP) or SES (rtTA and TetOn-GFP) and evaluated longitudinally by magnetic resonance imaging (MRI) or by histological staining at indicated time points. (**E**) Evaluation of tumor infection after 2 weeks from the LV injection by Nissl staining (left) and immunohistochemistry on coronal sections (50 μm of thickness) for GFP/V5 counterstained with DAPI. Quantification of the V5^+^ cells on the total number of DAPI nuclei in the SES-treated tumors is also shown (four tumors). *n* = 7 from four tumors. (**F**) Nissl staining of representative sections of the indicated models 5 weeks after the transplant and 4 weeks after the LV injection. (**G**) Quantification of tumor volume (five animals per group, means ± SEM): +dox: **P = 0.0046; −dox: *P* = 0.7731; statistically compared with unpaired *t* test. *n* = 5 from five tumors. Scale bars, 1 mm (B; E, left; and F) and 300 μm (E, right). WPI, weeks post infection.

We proceeded by assessing whether SES expression could repress tumor development in vivo. To more flexibly control SES activity, we used tetracycline-based (TetOn) inducible expression of the transgene for the in vivo experiments. This vector configuration was even more effective in eliminating CSCs in vitro than the constitutive vector (fig. S5, A to C). Next, we performed subcutaneous xenografts in nonobese diabetic (NOD)–severe combined immunodeficient (SCID) gamma (NSG) immunodeficient mice using patient-derived CSCs previously transduced with lentiviruses expressing either TetOn-GFP (mock) or TetOn-SES with or without doxycycline (dox) treatment (fig. S5D). Under the dox regimen, heterotopic transplants grew only from grafted mock cells, while SES-transduced cells were unable to sustain tumor growth (fig. S5, E and F), suggesting that this factor is toxic to glioma cells and prevents tumor formation. No or very few SES^+^ cells were identified when tiny human cell masses were isolated within the injection site, probably formed by not infected cells in vitro (fig. S5G). Next, orthotopic intracranial xenografts were carried out with GFP- or SES-expressing CSCs (Fig. 4A). Five weeks after brain transplantation, animals that did not receive dox-generated large tumor masses extending throughout the striatum (Fig. 4, Band C); however, even in this condition, tumors formed by SES-transduced cells were significantly smaller than those formed by control cells, indicating residual SES activity without dox induction (Fig. 4, B and C), as already ascertained in vitro (fig. S5A). Considering dox-treated animals, while mock GFP^+^-grafted cells formed evident tumor masses, grafted SES^+^ CSCs lost their tumor-initiating capacity, and no or extremely reduced tumors could be found in the transplantation site (Fig. 4, B and C). While mock-transduced cells formed GFP^+^ tumors without signs of cell death during their growth, SES-transduced cells were lost during the same time window, as evident by massive cleaved caspase-3 (CC3) staining, and the absence of V5^+^ cells, even in the residual masses of human cells, was eventually found (fig. S6A).

Although previous findings validated the antitumor activity of SES, the experimental design has no therapeutic relevance because it required an initial transduction of SES in cancer cells in vitro before grafting. Thus, we set up local SES viral transduction directly into a growing GBM mass in the brain parenchyma to curb its development. Intracranially transplanted CSCs were left to form a tumor mass for 7 days before injecting a mock (TetOn-GFP)–expressing or TetOn-SES–expressing lentivirus (Fig. 4D), and tumor growth was followed by weekly magnetic resonance imaging (MRI) T2-weighted scanning for histological analysis after 5 weeks. Both at 1 and 2 weeks after LV gene transfer, tumor masses were correctly transduced with either GFP (mock + dox) or SES (SES + dox), with a percentage of approximately 35% of V5^+^ cells found in SES-treated tumors (2 weeks after the infection) (Fig. 4E and fig. S6C). One week after viral treatment, SES-transduced tumors were enriched in dead cells and were depleted in mitotic figures that were rarely found in SES^+^ cells (fig. S6B). Four weeks after infection, mock-transduced tumors developed large tumors throughout the striatum (Fig. 4, F and G). In contrast, SES expression was sufficient to strongly reduce the tumor mass both at the end point (Fig. 4, F and G) and during the course of the treatment (fig. S6, B to D). These observations indicated that cancer cells transduced with the SES lentivirus were lost over time and that the remaining tumor tissue at 4 weeks from transplantation was composed exclusively of SES^−^ cells. Animals that did not receive dox developed masses with similar volumes under both conditions (Fig. 4, F and G), indicating that strong SES activity is required for tumor regression. Together, these data suggested that high levels of SES delivered by in situ viral treatment are able to markedly reduce the tumor mass in treated mice.

### SES eradicates tumorigenicity in mesenchymal SOX2^−^ GBM cancer cells

The mesenchymal glioblastoma state is the natural progression of recurring tumors mainly due to the treatments (e.g., irradiation and TMZ) and subsequent chronic brain inflammation (*15*, *16*, *30*, *31*). Mesenchymal tumors have been reported to exhibit less or none SOX2 dependency contrary to the other subtypes (*32*–*34*). Thus, we wondered whether SES treatment could also be effective against mesenchymal GBM cancer cells. To test this hypothesis, we first treated SOX2^−^ patient-derived CSCs established from a mesenchymal GBM tumor (*35*), finding them equally sensitive to SES antitumor activity (Fig. 4, A and B). Then, we used U87 glioma cells that have been widely used as a cell model of mesenchymal GBM (*35*, *36*) and displayed undetectable levels of SOX2 (fig. S7A). We found that SES expression in this cell type induced rapid cell proliferation loss (fig. S7, B and C). These results were mediated by global transcriptional deregulation with altered expression of key genes related to the cell cycle and apoptosis (fig. S7, D and E, and table S6). We reasoned that SES treatment may also be effective in SOX2^−^ cells because they probably maintain the expression of a large set of SOX2 target genes. Treated U87 and SNB19 cancer cells shared common deregulated SOX2 target genes, such as *MYC*, *JAG1*, *CDK6*, and *SEMA3A*, thus explaining the SES antitumor activity in U87 cells (fig. S7, G and H, and table S6). Notably, the concomitant overexpression of the full-length version of SOX2 did not hamper the SES function in this cellular system (fig. S7, B and C). We then sought to test the ability of SES to dampen the tumorigenic potential of U87 cells in vivo (fig. S7I). While heterotopic xenografts grew from grafted mock U87 cancer cells, SES-transduced cells were unable to sustain tumor growth (fig. S7, J to M), maintaining mouse survival (fig. S7N).

**Fig. 5.**
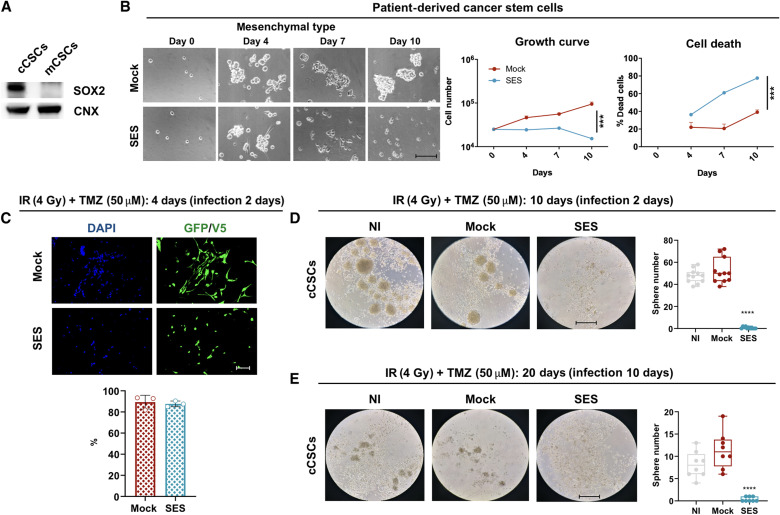
SES is active in mesenchymal CSCs. (**A**) WB for SOX2 and CNX (as loading control) in classic CSCs (cCSCs) and mesenchymal CSCs (mCSCs) confirm that our line of mCSCs is SOX2^−^. (**B**) Microphotographs, growth curve, and percentage of death cells (trypan blue automatic counting) of mCSCs. Growth curve: ***P = 0.0004; death cells: ***P = 0.006; statistically compared with two-way ANOVA. *n* = 3. (**C**) Immunocytochemistry for GFP and V5 tag, counterstained with DAPI in CSCs after 4 days of irradiation (IR) + TMZ and 2 days after GFP or SES viral infection. Quantification as percentage of GFP^+^ or V5^+^ cells on the total number of DAPI nuclei (means ± SEM). (**D**) Clones of mesenchymal shifted CSCs emerged after 10 days of IR + TMZ (infection at 2 days). Quantification as number of tumor spheres per well in either not infected (NI) cultures or GFP infected (mock) or SES infected (as boxplot shown as box for interquartile range, and median line and whiskers for highest and lowest values): GFP versus SES, ****P < 0.0001; statistically compared with one-way ANOVA. *n* = 11. (**E**) Clones of mesenchymal shifted CSCs emerged after 20 days of IR + TMZ (infection at 10 days after replating). Quantification as number of tumor spheres per well in either NI cultures or GFP infected (mock) or SES infected (as boxplot shown as box for interquartile range, and median line and whiskers for highest and lowest values): GFP versus SES, ****P < 0.0001; statistically compared with one-way ANOVA. *n* = 8. Scale bars, 300 μm (B), 100 μm (C), and 500 μm (D and E).

To be more strictly adherent to the pathological cell state changes occurring in GBM after standard treatment regimen, we set up two additional experimental settings. First, we treated both classical and proneural (both SOX2^+^) CSCs with tumor necrosis factor–α (TNFα), which was shown to be released by reactive astroglial cells in response to microglia activation (*37*) and to induce mesenchymal transition with strong SOX2 down-regulation (fig. S8, A to E). Notably, SES expression during TNFα treatment was able to reduce the growth of both CSC types (fig. S8, F to I). Furthermore, we used ionizing radiation and TMZ treatments as in the current clinical standard care that induced a SOX2^−^ mesenchymal shift in CSCs upon few days after irradiation and continuous drug administration (fig. S9, A to D). SES interfered with CSC development when delivered soon either after irradiation (Fig. 5, C and D, and fig. S9A) or after the full establishment of the mesenchymal cell phenotype (Fig. 5, C and E, and fig. S9A). These findings suggest that SES is also effective on GBM cancer cells with mesenchymal nature either as the original source of the tumors or as the result of an iatrogenic transition. Thus, SES effects are independent from SOX2 endogenous expression, probably by targeting plausibly the SOX2 downstream target genes with key roles in driving malignancy.

### SES is unharmful to healthy brain cells

**Fig. 6.**
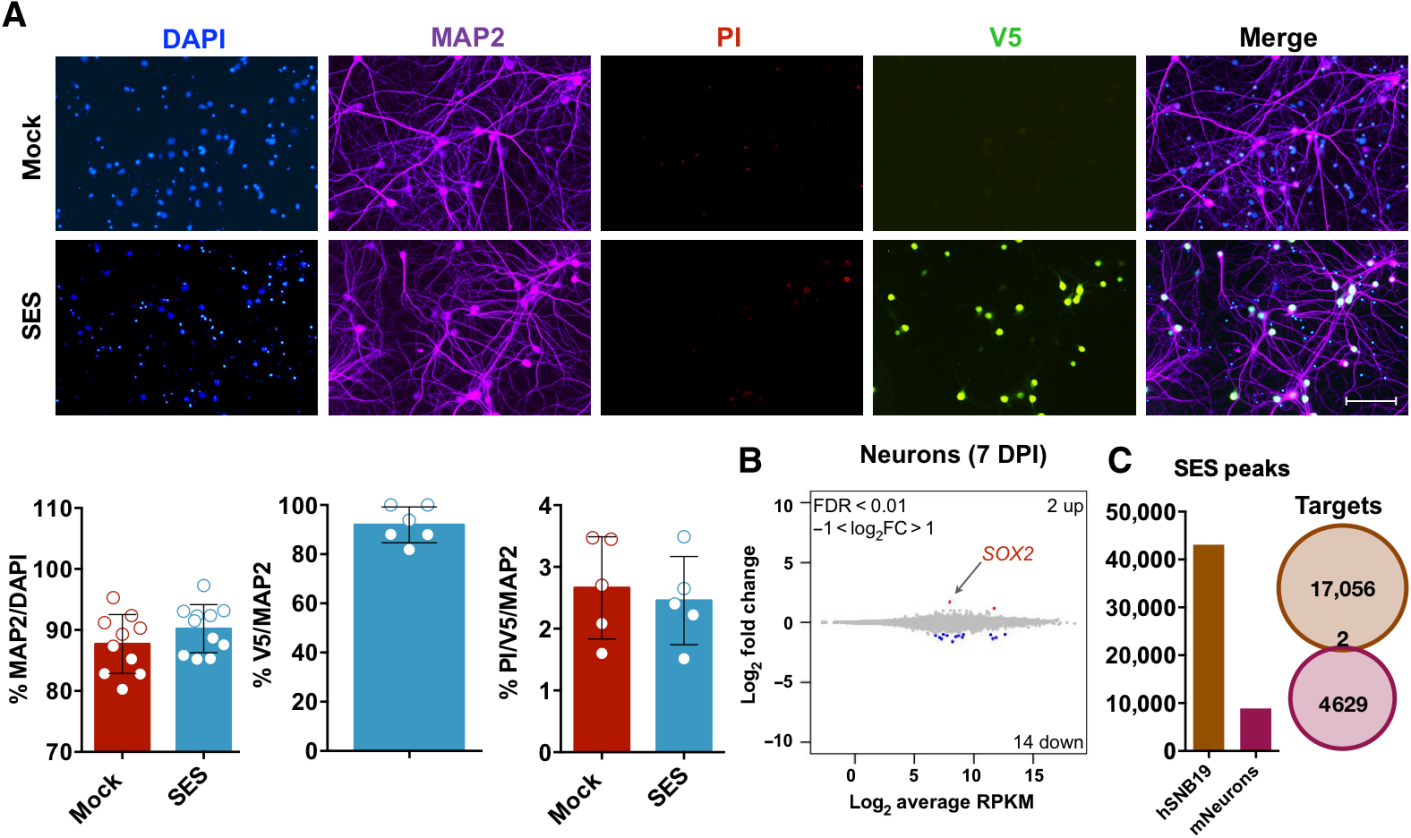
SES expression is unharmful in cultured neurons. (**A**) Immunocytochemistry with MAP2 (neuronal marker), PI (cell death), and V5 (tag) of primary murine hippocampal neurons 15 days after the infection with either mock or SES-expressing viruses, indicating no specific neurodegeneration induced by SES. Quantification (means ± SEM): MAP2 *P* = 0.2051 (*n* = 10); PI^+^/MAP2^+^ (or MAP2^+^V5^+^ in the case of SES): *P* = 0.6842 (*n* = 5); statistically compared with unpaired *t* test. (**B**) SES causes only marginal gene expression changes in mouse primary neurons as assessed by RNA-seq (7 DPI). (**C**) Quantification of CUT&Tag SES peaks in SNB19 cells (brown) and neurons (violet) (left). Venn diagram showing that the associated genes in SN19 and neurons are completely different (right). Scale bar, 100 μm (A).

**Fig. 7.**
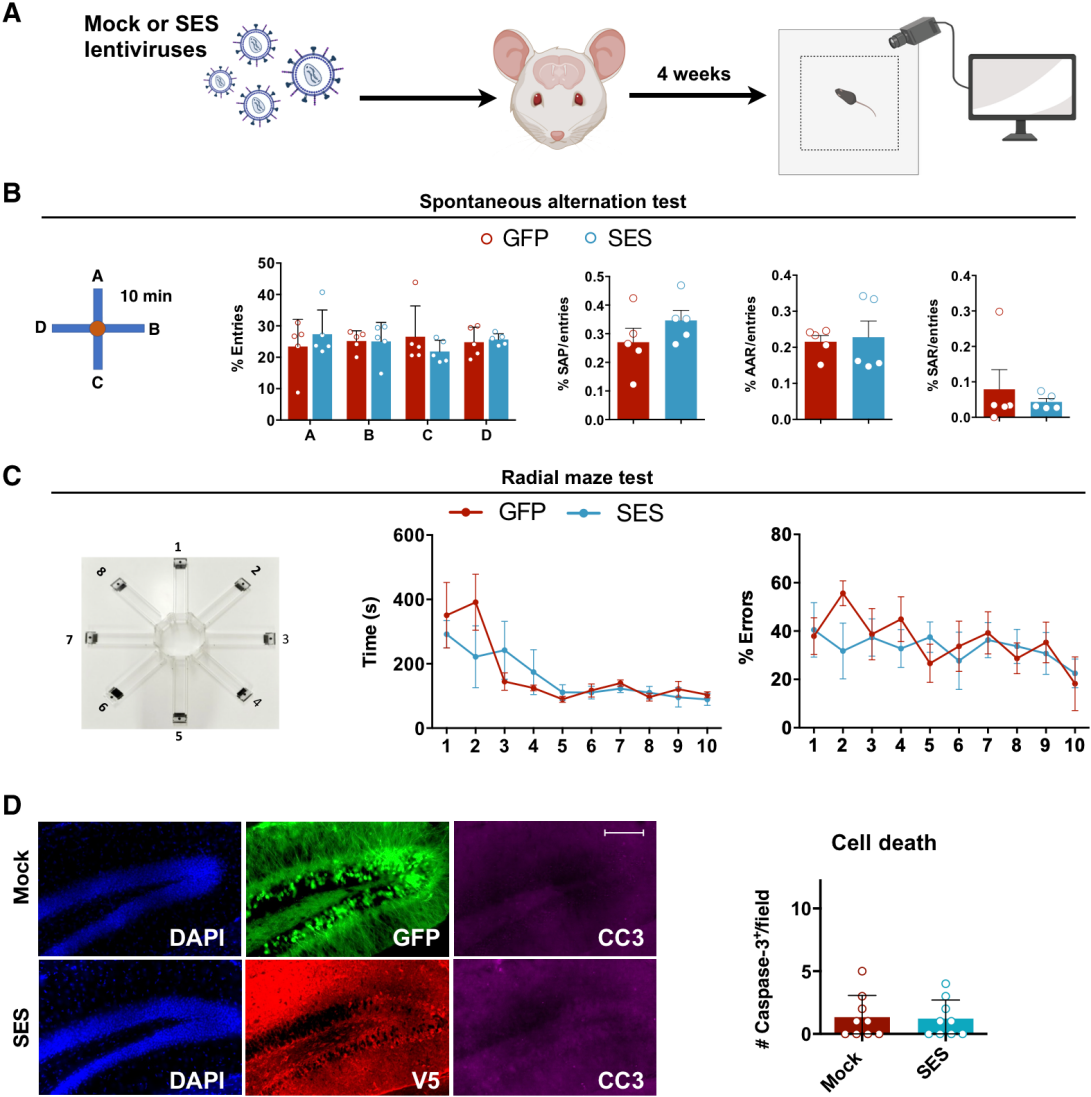
SES expression is safe in healthy brain. (**A**) Schematic representation of mock (GFP)/SES infection in hippocampi of wild-type (WT) mice followed by behavioral testing. (**B**) Spontaneous alternation test suggests no difference between mock- and SES-injected mice, as assessed by the percentage of the entries in the different arms and both the percentage of spontaneous alternation performance (SAP), the percentage of alternate arm return (AAR), and the percentage of same arm return (SAR) on the total entries. Quantification (means ± SEM): % entries: A, *P* = 0.8011; B, *P* > 0.9999; C, *P*= 0.6812; and D, *P* = 0.9990; statistically compared with two-way ANOVA. SAP: *P* = 0.3095; AAR: *P* = 0.9444; SAR: *P* = 0.9.444; statistically compared with Mann-Whitney test. *n* = 5 animals per group. (**C**) Radial maze test indicates no difference in the time to accomplish the task or tendency of SES-treated animals in committing errors during the entire protocol of the test compared to the mock-injected animals. Statistically compared with two-way ANOVA. *n* = 5 animals per group. (**D**) Quantification of CC3^+^ cells within infected hippocampi shows no difference between the conditions, indicating that SES is not toxic for murine neural cells. *n* = 9. Scale bar, 100 μm (D).

SOX2 is a pivotal factor in stem cells and neural progenitors, but it is strongly down-regulated during neuronal differentiation, with only a minority of mature brain cells expressing it in adulthood. To determine the effect of SES on healthy brain cells, primary mouse cortical neuronal cultures were treated with SES- or GFP (mock)–expressing LVs, and survival, morphology, and gene expression were assessed 2 weeks later. Mock- and SES-treated neurons displayed similar morphology with no sign of cell sufferance and with comparable low numbers of propidium iodide–positive (PI^+^) dying cells (Fig. 6A). Notably, the transcriptomes of mock- and SES-transduced cultures were substantially comparable, with only *Sox2* [RNA sequencing (RNA-seq) reads due to SES overexpression] and 15 other genes differentially expressed between the two neuronal populations (Fig. 6B and table S7). This may be due to the limited SES binding activity (Fig. 6C) caused by the heterochromatinization of the regions where the relative targets are located. Similar results were collected using human induced pluripotent stem cell (iPSC)–derived neuronal cultures, where SES treatment did not alter the survival and morphology of the MAP2^+^ neurons transduced (fig. S10, A and B). Next, we transduced SES (TetOn-SES + dox; for consistency with a previous tumor treatment experiment) into the hippocampus of adult C57BL/6 mice to assess hippocampal-dependent behavioral performance (Fig. 7A). Four weeks after stereotactic injections of mock (GFP) and SES LVs followed by dox treatment, both protein, DNA, and RNA of either transgene were equally detectable in the hippocampal tissue (fig. S10, C to E). Before autoptic analysis, mice were evaluated in the spontaneous alternation, radial maze, and Morris water maze tests to assess their exploratory behavior and cognitive function related to spatial learning and memory (Fig. 7, B and C, and fig. S10F). Both groups of animals performed equally well in these tasks, suggesting that SES expression did not elicit significant functional alterations in hippocampal neurons. Accordingly, comparable levels of cell death were found after the injections of GFP and SES LVs (Fig. 7D). For these experiments in murine cells, the original version of SES carrying the human SOX2_1–179_ was used (hSES), leaving open the possibility that, despite the extremely high similarity with the murine domain (eight different residues, all outside the DNA binding domain), its inactivity in murine neurons was due to a lack of functional conservation between mouse and human sequences. To investigate this hypothesis, we compared the activity of hSES and mSES (SES with murine SOX2 element) in both human and murine GBM cells (fig. S11). Either factor was delivered in cells with a vector in a Flex configuration, and after LV CRE expression, we found that the two SES versions were equally functional in both cell types (fig. S11), hence confirming the functional conservation of the SES orthologs in mouse and human cells.

**Fig. 8.**
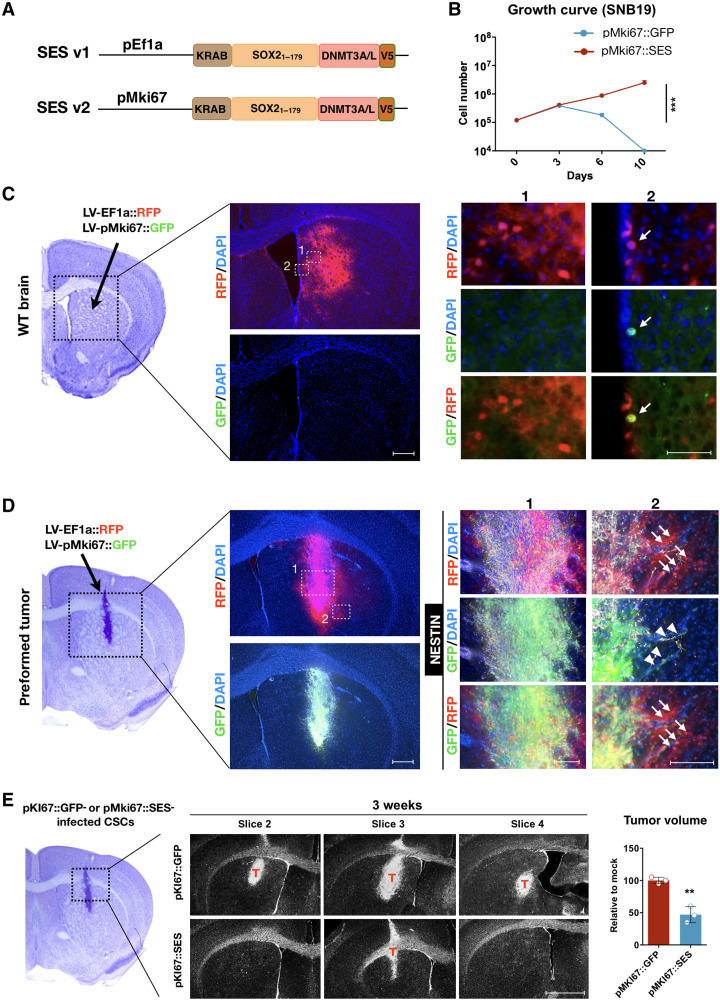
Restricted SES expression in mitotically active cells by the pMki67. (**A**) Scheme depicts the original SES (v1) and the further version (v2) carrying the *Mki67* promoter. (**B**) Growth curve of SNB19 cells: ***P = 0.0005; two-way ANOVA. *n* = 3. (**C**) Stereotactical injection in striatal region of WT mice of a mixture of LVs carrying either the Ef1α-RFP or the pMki67-GFP. After 1 week, the RFP protein is largely diffused in the striatum, while the expression of the GFP is confined in mitotically active neural progenitors lining the ventricles. Right: Magnification of the fields indicated in the images on the left. (**D**) Stereotactic injection in preformed tumors (CSCs injected 2 weeks before) within the striatal region of NSG mice with a mixture of LVs carrying either the Ef1α-RFP or the pMki67-GFP. After 1 week, both RFP staining and GFP staining are largely diffused in the tumor mass (human specific antibody: hNESTIN^+^). RFP^+^ cells (arrows) are also present in the mouse brain parenchyma (hNESTIN^−^), where the rare GFP^+^ (arrowheads) is infiltrating tumor cells (hNESTIN^+^), sustaining the specificity of the promoter. Right: Magnification of the fields indicated on the left. (**E**) DAPI-stained consecutive sections (400-μm distance between each pairs) of xenografts preinfected with either pMKI67::GFP or pMKI67::SES in NSG mice. T, tumor mass. Tumor volume quantification relative to the mean of the control (means ± SEM): *P* = 0.0024; unpaired *t* test. *n* = 3. Scale bars, 500 μm (C, left; D, left; and E) and 100 μm (C, 2; D, 1; and D, 2).

Regardless of these reassuring results, it cannot be excluded that SES-dependent chromatin changes can alter neuronal performance in vivo over longer periods of time, as occurs if this approach would be tailored for tumor treatment in humans. Thus, we conceived a strategy to restrict SES expression to cancer cells after viral inoculation into the brain. We isolated a 1.2-kb Mki67 gene promoter region (pMki67) (*38*) that was inserted into the LV cassette (SES v2) to drive SES expression exclusively in proliferative cells (Fig. 8A). First, we confirmed that pMki67 was driven high expression of GFP and SES in cancer cells (fig. S12A), and, consequently, pMki67-SES transduction significantly decreased the proliferation and survival of SNB19 cancer cells (Fig. 8B). Next, mouse primary neuronal cultures populated by both neurons and glial cells were transduced with either EF1α-GFP (constitutive) or pMki67-GFP LVs, and GFP distribution was investigated. Most MAP2^+^ neurons infected with pMki67-GFP did not express the transgene, while constitutive GFP was expressed in the entire cell population (fig. S12B). The few GFP^+^ cells in the pMki67-GFP–transduced cultures were also KI67^+^, likely corresponding to young proliferative astrocytes (fig. S12B), thus confirming the highly selective expression driven by pMki67. Thus, this strategy was effective in silencing SES expression in postmitotic brain cells without compromising SES transgene activation in proliferating cancer cells. Next, we injected a mixture of two lentiviruses expressing either red fluorescent protein (RFP) or GFP under the constitutive Ef1α or cell cycle–specific pMki67 into the striatum of normal animals (Fig. 8C). While RFP was present in a large fraction of the striatal parenchyma, GFP was detectable only in a few cells lining the lateral ventricles, presumably corresponding to dividing adult neural progenitors within the subependymal zone (white arrows in Fig. 8C). The same LVs were injected into a preformed tumor generated by grafting CSCs into the striatum 2 weeks earlier (Fig. 8D). Under this condition, GFP was widely detectable in the hNESTIN^+^ tumor mass (arrowheads in Fig. 8D), while it was excluded from the RFP^+^ healthy parenchyma (arrows in Fig. 8D). Last, we assessed whether SES expression driven by the pMKI67 promoter could repress tumor development in vivo. Orthotopic intracranial xenografts were carried out with CSCs preinfected with either pMKI67-GFP or pMKI67-SES (Fig. 8E). Three weeks after cell graft, animals that received pMKI67-SES–expressing cells generated smaller tumor masses compared to those derived from pMKI67-GFP CSCs (Fig. 8E). These data suggest that SES activity can be excluded from postmitotic brain cells by using the pMki67, enhancing the safety of the treatment but without affecting its efficacy against cancer cells.

## DISCUSSION

The activity of developmental TFs is mainly restricted to morphogenesis, when these TFs play a prominent role in stem cell identity, cell lineage commitment, and differentiation. However, these TFs can be reactivated or hijacked by the cancer genetic program to propel tumor development and progression. It is estimated that approximately 20% of all known oncogenic proteins are represented by TFs (*39*) with critical importance for acquiring malignant behaviors of dedifferentiation, proliferation, and migration. Despite the extensive role of these TFs in tumors, interfering with their functions from a translational perspective has proven challenging. Despite some notable success (*40*), stable and complete gene silencing by various genetic tools or small molecules has been arduous to achieve in cancer cells. Moreover, the cancer genetic program has been repeatedly shown to overcome inactivation of single genes by reconfiguring the transcriptional network to promote cancer resistance and recurrence. Here, we designed an epigenetic repressor (SES) by rational reconfiguration of SOX2 through the assembly of transcriptional and epigenetic negative regulators of gene transcription. Both epigenetic elements are required to sustain SES antitumor activity, which is erased by preventing the binding of the HMG-box domain to the DNA. This design is modular and versatile and can be, in principle, applied to many other activating oncogenic TFs. SES-dependent de novo DNA methylation in SOX2 target genes triggered by the DNMT3A/L catalytic domains promoted chromatin closure and stable silencing of the SOX2 downstream network. Thus, SES-dependent gene repression is achieved by both histone modification and widespread DNA methylation, which should ensure long-term and irreversible gene silencing even if SES is expressed only transiently. These wide transcriptional changes inhibited the proliferation of cancer cells that failed to cope with these alterations, triggering diffuse cell death. These repressor domains were previously associated with TALEN or catalytically dead Cas9 nucleases to generate synthetic transcriptional repressors (*22*, *23*). Here, we show that these domains can reconfigure the transcriptional activity of endogenous TFs while preserving their chromatin occupancy and target selectivity.

SOX2 expression is fundamental to control self-renewal and malignant phenotypes in GBM cancer cells as well as in many stem cells, including pluripotent and neural types. We demonstrated that SES maintains its efficacy to silence the SOX2 target genes that support the proliferative capability and apoptosis resistance of cancer cells, even when SOX2 itself is not expressed. Thus, dominant negative activity of SES can also be detrimental to GBM subtypes or subclones that develop SOX2 independence, as, for instance, in the mesenchymal transition occurring upon current clinical treatments (*15*–*18*), in which an approach based on simple *SOX2* knockdown is ineffective. SOX2 has a primary role in promoting tumor development in many malignancies other than GBM, including medulloblastoma and lung, prostate, and breast cancers (*41*–*44*). Hence, the use of SES or other epigenetic silencing factors (ESFs) can be expanded for the treatment of other cancers. Tumor targeting of ESFs by viral-mediated delivery can, in principle, be effective in cancers contained within solid tissues that can be efficiently targeted by viral transduction in vivo. In this scenario, cancers in the liver, lung, breast, and kidney might be plausible targets for this approach, because delivery routes and viral strains are known to obtain specific and high tissue transduction efficiency. Similarly, the same approach can be proposed to treat metastatic masses in these same organs.

Here, SES was directly injected into the tumor mass to repress its growth. A similar approach can be useful in a clinical setting to treat glioblastoma whose surgical resection is impracticable because of unattainable locations within the brain or close proximity to vital brain regions. Moreover, SES can be delivered by viral transduction in the brain parenchyma surrounding the resected primary tumor as adjuvant therapy to target the remaining cancer cells and restrain subsequent tumor recurrence.

Here, glioblastoma treatment with SES was carried out through LV transduction by local injections into the affected tissue. However, alternative therapeutic viruses could be similarly used as, in particular, specific engineered strains of adeno-associated viruses that can spread much better than lentiviruses throughout the brain tissue due to their small size. Maximizing viral spreading in the brain parenchyma increases the targeting efficiency of cancer cells scattered in the tissue, providing better protection against tumor recurrence. Moreover, nonviral vehicles such as nanoparticles or liposomes might be considered to deliver SES mRNA or protein to obtain acute transgene expression, which can still be sufficient to inhibit cancer cells while strongly enhancing the overall safety profile of the entire procedure.

Although we provided solid evidence that SES expression is unharmful to neuronal cultures and, at least in the short/medium term, in the murine brain, we developed a strategy to restrict its activation only to proliferative cells, which are strongly enriched in cancers and rarely present in the brain parenchyma. This feature is of high importance because, in the initial design, the transgene was expressed under a constitutive promoter, and its effect over a long period of time remained unpredictable. The pMki67 was highly effective in expressing the viral transgene in cancer cells but not in parenchymal brain cells.

In summary, we configured an epigenetic repressor that operates as a dominant negative version of the oncogenic SOX2 TF and is able to bind and stably repress components of the SOX2 transcriptional network. Targeted viral delivery of SES in glioblastoma is sufficient to inhibit tumor development by blocking cell proliferation and inducing cell death. Given its wide applicability to other oncogenic TFs and the high efficiency of targeting cancer cells by viral transduction, this approach offers an innovative strategy to build antitumor molecular tools effective against glioblastoma and other deadly cancers.

## MATERIALS AND METHODS

### Constructs

#### KRAB-hSOX2

The full-length human *SOX2* gene was fused with the KRAB repressor domain (from the gene *ZNF10* encoding for a zinc finger protein; amino acids 1 to 97) at its N-terminus (term), while V5 tag was fused at the C terminus of the SOX2 domain. The transgene was used in an LV construct, with Ef1α as promoter.

#### KRAB-hSOX2-D3A&L

The full-length human *SOX2* gene was fused with the KRAB repressor domain (from the gene *ZNF10* encoding for a zinc finger protein; amino acids 1 to 97) at its N-term, while the functional domains of DNMT3A (amino acids 388 to 689) and DNMT3L (amino acids 206 to 421) were fused at the C terminus of the SOX2, and V5 tag was fused at the end of the last domain, at the C terminus of the new chimeric transgene. The transgene was used in an LV construct, with Ef1α as promoter.

#### KRAB-hSOX2^1–179^

The initial part of human *SOX2* gene, coding for amino acids 1 to 179 (thus excluding the SOX2 activator domain), was fused with the KRAB repressor domain (from the gene *ZNF10* encoding for a zinc finger protein; amino acids 1 to 97) at its N-term, while V5 tag was fused at the C terminus of the SOX2. The transgene was used in an LV construct, with Ef1α as promoter.

#### hSOX2^1–179^-D3A&L

The initial part of human *SOX2* gene, coding for amino acids 1 to 179 (thus excluding the SOX2 activator domain), was fused with the functional domains of DNMT3A (amino acids 388 to 689) and DNMT3L (amino acids 206 to 421) and V5 tag at the C terminus of the SOX2. The transgene was used in an LV construct, with Ef1α as promoter.

#### SES v1

The initial part of human *SOX2* gene, coding for amino acids 1 to 179 (thus excluding the SOX2 activator domain), was fused with the KRAB repressor domain (from the gene *ZNF10* encoding for a zinc finger protein; amino acids 1 to 97) at its N-term, while the functional domains of DNMT3A (amino acids 388 to 689) and DNMT3L (amino acids 206 to 421) were fused at the C terminus of the SOX2 portion, and V5 tag was fused at the end of the last domain, at the C terminus of the new chimeric transgene. The transgene was used in an LV construct, with Ef1α as promoter.

#### KRAB-hNEUROD1-D3A&L

The initial part of human *NEUROD1* gene, coding for amino acids 1 to 153 (thus excluding the NEUROD1 activator domain), was fused with the KRAB repressor domain (from the gene *ZNF10* encoding for a zinc finger protein; amino acids 1 to 97) at its N-term, while the functional domains of DNMT3A (amino acids 388 to 689) and DNMT3L (amino acids 206 to 421) were fused at the C terminus of the NEUROD1 portion, and V5 tag was fused at the end of the last domain. The transgene was used in an LV construct with Ef1α as promoter.

#### SES (R74P/L97P)

SES construct was mutated in residues 74 and 97 of the initial part of human *SOX2* gene (arginine in position 74 and leucine in position 97 to two prolines).

#### TetOn-SES

The transgene was the same of the SES version 1, and the Ef1α promoter was replaced with a tetracycline-dependent promoter. Its expression needs reverse tetracycline-controlled transactivator (rtTA) protein and the dox.

#### SES v1.1

The transgene was the same as version 1, and the Ef1α promoter was replaced with the proximal promoter of the murine *Mki67* gene (−1263 to −1 related to *Mki67* atg).

#### Flex-SES

The SES transgene (as for the SES v1) was inserted between two tandem of LoxP sites (LoxP and Lox2272; see fig. S3) (*28*) in reverse direction in an LV vector with Ef1α as promoter. CRE recombinase is necessary for transgene flipping into the correct orientation for productive expression.

#### Flex-mSES

To generate this murine version, in the Flex-SES, the human SOX2 domain was substituted with the equivalent murine coding for amino acids 1 to 181 of the *Sox2* sequence.

### Lentivirus production

LV replication–incompetent, vesicular stomatitis virus glycoprotein–coated LV particles were packaged in 293T cells (*45*). Cells were transfected with 30 μg of vector and packaging constructs, according to a conventional CaCl_2_ transfection protocol. After 30 hours, the medium was collected, filtered through 0.44-μm cellulose acetate, and centrifuged at 20,000 rpm for 2 hours at 20°C to concentrate the virus. Each viral batch was titrated with a dedicated kit (MoBiTec, number CBTDL10900). The titer obtained is in IFU/ml (infections units per milliliter), according to the manufacturer, and the amount of genomic RNA in a sample can be converted to its viral titer by calibration with the standards of LV preparations provided. The viral batch was used when titer is 10^8^ IFU/ml.

### Cell cultures

U87, U251, and SNB19 (human glioblastoma cell lines) were cultured in plastic adherence condition in DMEM (Dulbecco’s modified Eagle’s medium; high glucose; Sigma-Aldrich) containing 10% fetal bovine serum (FBS; Sigma-Aldrich), 1% penicillin/streptomycin (Pen/Strept) (Sigma-Aldrich), 2 mM glutamine (Sigma-Aldrich), 1% nonessential amino acids (Thermo Fisher Scientific), and 1% sodium pyruvate solution (Sigma-Aldrich). GL261 (mouse glioblastoma cell line) was cultured in plastic adherence condition in DMEM (high glucose; Sigma-Aldrich) containing 10% FBS (Sigma-Aldrich), 1% Pen/Strept (Sigma-Aldrich), and 2 mM glutamine (Sigma-Aldrich). All the cell lines were passaged twice a week using a trypsin-EDTA solution (Sigma-Aldrich). CSCs (gift of R. Galli) from classical (L0627), mesenchymal (L1312), and proneural (L0512) glioblastoma tumors were maintained in spheres in suspension cultures in DMEM/F12 (Sigma-Aldrich) supplemented with hormone mix {DMEM/F12, 0.6% glucose (Sigma-Aldrich) [30% in phosphate-buffered saline (PBS) (Euroclone)], insulin (250 μg/ml) (Sigma-Aldrich), putrescine powder (97 μg/ml) (Sigma-Aldrich), apo-transferrin powder (Sigma-Aldrich), 0.3 μM sodium selenite, and 0.2 μM progesterone}, 1% Pen/Strept, 2 mM glutamine, 0.66% glucose [30% in PBS (Euroclone)], and heparin (4 mg/ml; Sigma-Aldrich); basic fibroblast growth factor (20 ng/ml; Thermo Fisher Scientific) and epidermal growth factor (20 ng/ml; Thermo Fisher Scientific) were freshly added to culture medium. Sphere cultures were passaged twice a week by mechanical dissociation of the sphere to a single-cell suspension. Dox (2 μg/ml) was added when appropriated. CSCs, when needed, were treated as previously described (*30*) with TNFα (10 ng/ml) (PeproTech, 300-01A) starting at day 0 for the whole duration of the experiment. After 2 or 5 days, cells were infected and seeded in 24-well plates. Stable cell lines were generated by selecting infected positive cells with puromycin (1 μg/ml) (Sigma-Aldrich) added in the culture medium 2 days after infection. All the cultures were kept in a humidified atmosphere of 5% CO_2_ at 37°C under atmospheric oxygen conditions. A list of the cell models used in this study is available in table S8.

### Cell growth analysis

A total of 3 × 10^5^ to 5 × 10^5^ of cancer cell lines were seeded in adherent condition in a six-multiwell plate at day 0; at day 1, cultures were infected with LV vectors (10 μl per well) and, at day 3, cells were detached, and live cells were stained with trypan blue solution (0.4%; Thermo Fisher Scientific) and counted using the Countess II Automated Cell Counter (Thermo Fisher Scientific); after this passage, 3 × 10^5^ to 5 × 10^5^ cells were seeded again. This was repeated for three time points every 3 to 4 days; the experiment was repeated three times for each time point. Bright-field representative pictures were taken at each time point.

A total of 2.5 × 10^4^ to 5 × 10^4^ CSCs were infected with LV vectors (expressing either SES or GFP; 2 μl per well) and seeded in a single-cell suspension condition in a 24-multiwell plate at day 0. At days 4, 7, 10, and 13, CSC spheres were dissociated to a single-cell suspension mechanically or using Accutase solution (Sigma-Aldrich), and live cells were stained with trypan blue solution and counted as previously described. Live cell number and the percentage of dead cells were reported on graphs for each time point; the experiment was repeated three times for each time point. Bright-field representative pictures were taken at each time point. Replicates represent the number of cells counted in independent platings.

### Western blot analysis

Cells were homogenized in radioimmunoprecipitation assay buffer {50 mM tris (pH 7.5), 150 mM NaCl, 1 mM EDTA, SDS [0.1% for cells and 1% for three-dimensional (3D) cultures], 1% Triton X-100, Roche Complete EDTA-free Protease Inhibitor Cocktail, and Roche PhosSTOP EASYpack}, and Western blot analysis was performed incubating primary antibodies overnight at 4°C in blocking solution composed of 5% bovine serum albumin (Sigma-Aldrich) or 5% nonfat dry milk in 0.1% PBS-Tween 20 (Sigma-Aldrich) according to antibody datasheet. The primary antibodies used were as follows: anti-V5 (1:1000; mouse; Thermo Fisher Scientific, R96025), anti-SOX2 (1:500; clone no. 245610, mouse; R&D Systems, MAB2018), anti–histone H3 (1:2000; rabbit; Abcam, ab1791), anti-CD44 (1:1000; rabbit; Cell Signaling Technology, 37259), anti-NDRG1 (1:1000; clone D6C2, rabbit; Cell Signaling Technology, 9408), anti–signal transducer and activator of transcription 3 (STAT3) (1:1000; clone 124H6, mouse; Cell Signaling Technology, 9139), anti-pSTAT3 (1:2000; Tyr^705^, clone D3A7, rabbit; Cell Signaling Technology, 9145), anti-calnexin (1:2000; rabbit; Sigma-Aldrich, C4731), anti–glyceraldehyde-3-phosphate dehydrogenase (1:10,000; mouse; Abcam, mAB9484). Antibodies used in this work are listed in table S9. Band densitometry relative to control was calculated using Fiji software (National Institutes of Health, USA), normalized on housekeeping genes.

### Clonogenic assay

A total of 2.5 × 10^4^ CSCs were infected or not with LV vectors (2 μl per well, expressing either SES or GFP) and seeded in a single-cell suspension condition in a 24-multiwell plate at day 0. At day 6, spheres were dissociated to a single-cell suspension and live cells were counted as previously described, and 2.5 × 10^4^ to 5 × 10^4^ CSCs were seeded again, letting cells grow and form spheres until day 10. Bright-field images were taken at days 6 and 10, and the resulting number of spheres was counted for each condition (not infected, GFP-infected, or SES-infected); sphere diameter was measured and the percentage of sphere having a diameter of <100 mm was reported on the graph for each condition and time point. The experiment was repeated three times for each time point. Replicates showed in the bar graphs represent the number of spheres counted in independent platings.

### Cell death assay by flow cytometry

Cell death was estimated by flow cytometry. Briefly, cells were detached using trypsin (SNB19), or spheres were disaggregated using Accutase solution (Sigma-Aldrich). SNB19 cells treated with staurosporine (2 μm for 12 hours; Sigma-Aldrich) were added as positive control for cell death. Approximately 1 × 10^6^ cells were incubated using a Zombie Aqua fixable viability kit (BioLegend, 423101) diluted 1:100 in PBS at room temperature, in the dark, for 15 min. After incubation, cells were washed using PBS by centrifugation for 5 min at 500*g*. Samples were then immediately acquired (approximately 10,000 events per sample), and the fluorescence level was measured using a CytoFLEX (Beckman Coulter) fluorescent cell analyzer. Data were analyzed using the FCS Express 7 (De Novo Software).

### Irradiation and TMZ treatment

CSCs were treated as previously described (*30*), with 50 μM TMZ starting 2 hours before irradiation and for the whole duration of the experiment. Control cells were treated with same doses of dimethyl sulfoxide (Sigma-Aldrich, D2650). Irradiation was delivered using the Biobeam GM 2000 (Gamma Service) platform at a dose of 4 gray (Gy). After 2 or 10 days, cells were infected and seeded in 24-well plates. Replicates showed in the boxplots represent the number of spheres counted in independent platings.

### In silico modeling

To identify the best domain configuration and to model the SOX2 epigenetic repressor, we generated different fusion proteins with the SOX2 transgene located at the N terminus (S_K_D), at the center (SES), or at the C terminus (D_K_S) of the protein. Every single sequence was entirely submitted to I-TASSER (*24*), a 3D structure predictor, based on the homology modeling algorithm. From the software, output was kept, and for each protein configuration, the best quality model of the top five was ranked by I-TASSER score (C-score) for further analysis. SES version was chosen on the basis of the C-score and root mean square deviation of atomic positions that allow to know the general folding rate of a protein structure (table S1). SES first ranked model was used to generate the quality check of the structure such as normalized B-factor, coverage, and residue-specific quality (table S1).

To assess whether the ability to bind DNA is maintained in SES, a blind docking simulation analysis, using Haddock (*46*), was performed with the obtained predicted SES model and a double helix DNA strand. The DNA used as ligand was extracted from the Protein Data Bank file 1O4X (the ternary complex of the DNA binding domains OCT1 and SOX2 TFs with a 19-bp oligonucleotide). The top 10 poses ranked by 𝚫*G* of binding (kcal/mol) were used for further analysis (table S1).

### RNA isolation and real-time reverse transcription quantitative polymerase chain reaction

RNA was extracted using the TRIzol reagent isolation system (Sigma-Aldrich) according to the manufacturer’s instructions. For quantitative reverse transcription polymerase chain reaction (qRT-PCR), 1 μg of RNA was reverse-transcribed using the ImProm-II Reverse Transcription System (Promega); thereafter, qRT-PCR was performed in triplicate with custom-designed oligos using the CFX96 Real-Time PCR Detection System (Bio-Rad, USA) using the Titan HotTaq EvaGreen qPCR Mix (Bioatlas). Obtained cDNA was diluted 1:10 and was amplified in a 16-μl reaction mixture containing 2 μl of diluted cDNA, 1× Titan Hot Taq EvaGreen qPCR Mix (Bioatlas, Estonia), and 0.4 mM of each primer. Analysis of relative expression was performed using the ΔΔC_t_ method, using 18*S* ribosomal RNA as a housekeeping gene, and using the CFX Manager software (Bio-Rad, USA). The viral genome quantity was estimated using qPCR on DNA using primers on a portion of LV vector (common for GFP and SES) and on murine nuclear DNA as normalizer. Primers used in this work are listed in table S10.

### Digital droplet qPCR for copy number determination assays

A total of 2.5 × 10^6^ CSCs were infected with LV vectors (either SES or GFP) and seeded in a single-cell suspension condition in a six-well plate. At days 2 and 7 after infection, samples were collected. RNA was extracted using the RNeasy Mini Kit (QIAGEN, catalog no. 74104) according to the manufacturer’s protocol. One microgram of RNA was reverse-transcribed to generate cDNA, as previously described. DNA was extracted using the NucleoSpin DNA RapidLyse (Macherey-Nagel, REF 740100.50) according to the manufacturer’s protocol. Droplet Digital PCR (ddPCR) was performed using a QX100 Droplet Digital PCR System (Bio-Rad). Reaction mixture (22 μl) contained 2× ddPCR Master Mix (Bio-Rad), 900 nM of each PCR primer, 250 nM hydrolysis probes, and 5 ng of genomic DNA (gDNA) samples (L0627 CSCs LV-GFP or LV-SES infected at 2 and 7 DPI, stable line L0627 CSCs hFLEX-SES LV-CRE infected at 2 and 7 DPI) or 1 ng of starting RNA for cDNA samples (L0627 CSCs LV-GFP or LV-SES infected at 2 and 7 DPI, stable line L0627 CSCs hFLEX-SES LV-CRE infected at 2 and 7 DPI). The sequences of the primers and probes for woodchuck hepatitis virus (WHV) posttranscriptional response element (WPRE) target and for hLMNB2, used as reference, are reported in table S10. Each 20 μl of PCR reaction was dispersed in a water-in-oil emulsion to generate droplets using the QX100 Droplet Generator (Bio-Rad); a final volume of 40 μl of droplet-partitioned samples, containing approximately 20,000 droplets, was generated for each sample. Thermal cycling conditions were 95°C for 10 min followed by 45 cycles of 94°C for 30 s and 60°C for 1 min; the ramp rate for the entire run was 2°C/s. After thermal cycling, the PCR plates were transferred to the QX100 Droplet Reader (Bio-Rad) for reading and counting positive and negative droplets. The average number of droplets read for each ddPCR was 20612 (SD:532). The concentration of the target fragment (copies/μl) for cDNA or the copy number for gDNA (concentration of target fragments normalized on reference fragments) and the relative standard uncertainty were calculated on the basis of the Poisson distribution using the QuantaSoft software (*47*).

### RNA-seq and analysis

RNA libraries were generated starting from 1 mg of the total RNA (deriving from U87, SNB19, and murine hippocampi), which quality was assessed by using a TapeStation instrument (Agilent). To avoid overrepresentation of 30 ends, only high-quality RNA with RNA integrity number of >8 was used. RNA was processed according to the TruSeq Stranded mRNA Library Prep Kit protocol. The libraries were sequenced on an Illumina HiSeq 3000 with 76-bp stranded reads using Illumina TruSeq technology. Image processing and base call were performed using the Illumina Real-Time Analysis Software. FASTQ files were aligned to hg38 or mm10 human or mouse reference genomes by using the splice junction map per TopHat (*48*). Differential gene expression and functional enrichment analyses were performed with DESeq2 (*49*) and gene set enrichment analysis (*50*), respectively.

### ChIP sequencing

Chromatins were isolated from SNB19 (two biological replicate for each condition). Cells were plated in adherent condition using Matrigel-coated 15-mm plates at a density of 6 × 10^6^ per plate. When the plates reached 90% of confluence, cells were fixed, adding formaldehyde directly to the cell culture medium to reach a final concentration of 1%, and were incubated for 10 min at room temperature. The reaction was quenched, adding glycine to a final concentration of 125 mM and incubated 5 min at room temperature. The medium was then removed, and cells were washed three times with cold, sterile PBS + protease inhibitors, and cells were gently scraped and collected for centrifugation at 4°C for 50 at 1200 rpm. ChIP experiments were performed as previously described (*51*). Briefly, collected cell pellets were lysed in lysis buffer [50 mM tris-HCl (pH 8), 0.1% SDS, 10 mM EDTA (pH 8), 1 mM phenylmethylsulfonyl fluoride (Sigma-Aldrich, no. P7626), and protease inhibitor cocktail (Roche, #04693159001)], and chromatin was sonicated with a Branson D250 sonifier (four cycles of 30 s, 20% amplitude) to reach an average fragment size of 0.1 to 0.5 kb. Following quantification, 100 mg of sonicated chromatin was used in each immunoprecipitation and incubated O/N (overnight) at 4°C with 4 mg of V5 antibody (1:5; mouse; Thermo Fisher Scientific, R96025).

ChIP-seq libraries were produced using 5 ng of each immunoprecipitated and purified DNA. End repair of DNA fragments was achieved by sequential 15-min incubations at 12° and 25°C with T4 polynucleotide kinase (PNK) (0.15 U/ml) [New England Biolabs (NEB), #M0201L], T4 polymerase (0.04 U/ml ) (NEB, #M0203L), and 0.1 mM deoxynucleotide triphosphates (NEB, #N0446S). A base addition was performed by an incubation with Klenow fragment (0.25 U/ml) (NEB, # M0212L) and 167 mM 2′-deoxyadenosine 5′-triphosphate (NEB, #N0440S) for 30 min at 30°C. Adaptor ligation was achieved by using the Quick Ligation Kit (NEB, #M2200L) and by performing an incubation of 15 min at 25°C. DNA fragments were lastly amplified for 14 cycles, by using the PfuUltra II Fusion HS DNA polymerase kit (Agilent, #600674). DNA purification steps after each enzymatic reaction were performed using Agencourt AMPure XP SPRI beads (Beckman Coulter, #A63882). The obtained libraries were quality controlled using an Agilent Bioanalyzer (Agilent Technologies, #G2943CA) before sequencing with Illumina HiSeq 2000.

### CUT&Tag sequencing

CUT&Tag was performed according to a previously published protocol (*29*). Briefly, after obtaining a single-cell suspension for each experimental condition, cells were counted, and 100,000 cells were used for each experimental replicate (three biological replicates for each condition). Afterward, nuclei are extracted, light-fixed with 0.1% formaldehyde, bound to concavalin beads, and then incubated overnight with a primary antibody (V5, Abcam, ab15828) or control antibody (rabbit, immunoglobulin G). The next day, nuclei suspensions are incubated with secondary antibody and washed, and fragmentation of DNA is performed using protein A-Tn5 conjugates (Diagenode, C01070001). DNA is then released from the nuclei, and sequencing libraries are amplified using a single-indexed barcode according to a previously published protocol (*52*). Last, each individual library has been paired-end sequenced on an Illumina NovaSeq platform.

### MeDIP sequencing

On microgram of purified gDNA was used with a QIAamp DNA mini kit, two biological replicates for each condition (QIAGEN, catalog no. 51304). Briefly, for methylated DNA immune precipitation and purification, a MagMeDIP-seq kit was used (Diagenode, code C02010040). First, gDNA was sonicated to obtain a fragment size between 150 and 300 bp, and then it was denaturated to single-stranded DNA and immune-precipitated using an anti-methylcytosine antibody provided by the kit. The next day, immunoprecipitated DNA and input were purified and eluted. Library preparation was performed using the NEBNext Ultra II kit for Illumina (code E7645), following the manufacturer’s instruction. Each library was dual-indexed using NEBNext Multiplex Oligos for Illumina (code E6440) and sequenced at 30 million pair-end depth with Illumina HiSeq 2000.

### ATAC sequencing

ATAC-seq was performed using 50,000 cells for each experimental condition (SNB19 infected with SOX2V5 or SESV5), two biological replicates each. Briefly, cells were detached, counted, collected, and then washed by performing a centrifuge at 500 rcf (Relative Centrifugal Force) for 5 min at 4°C in PBS. Subsequently, cells were gently lysed by suspending them in 50 μl of ATAC–RSB (resuspension buffer) (*52*) supplemented with 0.1% NP-40, 0.1% Tween 20, and 0.01% digitonin for 5 min on ice. After the lysis, cells were washed using 1 ml of ATAC-RSB with Tween 20, but with no NP-40 or digitonin. Transposition was then performed using Tn5 transposase and buffer from Illumina (code 20034197). Transposition buffer was complemented with 0.1% digitonin and 10% Tween 20 to have a better yield in transposition reaction. After transposition, DNA was purified, and libraries were amplified for the required amount of cycles as previously described (*53*). Each replicate was sequenced at a depth of 50 million paired-end reads on Illumina NovaSeq.

### Bioinformatics analysis

Sequenced reads were quality-checked and adaptor-trimmed with FastQC (www.bioinformatics.babraham.ac.uk/projects/fastqc/) and Trimmomatic (*54*). Reads were aligned to the hg19 human genome using Bowtie2 version 2.2.3 (*55*). Only uniquely mapped reads were used in the subsequent analyses, with an average mappability of >96% of the initial total reads. Peak calling was performed with MACS3 (*56*), with the following parameters: --broad -g hs --broad-cutoff 0.1 --nomodel --extsize 50 --down-sample -q 0.2. Peak annotation [±10-kb TSS (Transcription Start Site)] was performed with ChIPseeker (*57*) and TxDb.Hsapiens.UCSC.hg19.knownGene (*58*). Functional enrichment analysis of putative targets was performed with GeneSCF (*59*). Normalized BigWig tracks, coverage matrix calculation, heatmaps, and enrichment profiles were produced with DeepTools2 (*60*). Specifically, coverage was computed by computeMatrix, with the following parameters: --averageTypeBins median --downstream 1000 --upstream 1000 -- skipZeros --smartLabels --sort_Reg_ions descend --sortUsing median. Coverage heatmaps were made with plotHeatmap, with the following parameters: --kmeans 3 --plotType lines --sortRegions descend --sortUsing median --averageTypeSummaryPlot median --zMax 20. Coverage profiles were made with plotProfile, with the following parameter: --averageType median. Data of ESC and NPCs SOX2 ChIP-seq are publicly available through GSE69479 (*61*). Genomic data generated in this study are accessible through the Gene Expression Omnibus (GEO) database with the GEO series accession number GSE200062.

### Human xenografts in mice

GBM lines or CSCs L0627 were seeded in six-well dishes and infected with 10-μl LV-EIF1α-SES or LV-EIF1α-GFP, or EIF1α-rtTA + TetON-GFP or EIF1α-rtTA + TetON-SES (10^8^ IFU/ml) for each well for 48 hours.

#### Heterotopic xenografts

A total of 3 × 10^6^ infected cells were suspended in 100 μl of Matrigel (Matrigel growth factor reduced, Corning). By using 1-ml syringes previously cooled at 20°C, GFP-infected cells were subcutaneously injected into the left flank of NSG mice (NOD.cg-Prkdc scid Il2rg tm1Wjl/SzJ), whereas SES-infected cells were subcutaneously injected into the right flank of the same animal. Mice were euthanized at 1 to 3 months after injections (according with growth rate), and subcutaneous growing tumors were extracted and fixed in 4% paraformaldehyde (PFA) for, at least, 24 hours. Tumor samples were sized and kept O/N in 30% sucrose in PBS and then embedded in optimal cutting temperature (O.C.T.) for cryopreservation. Histological slides were cut in 50-μm sections on cryostat (CM1850 UV, Leica). Subsequently, the sections were processed for immunofluorescence or mounted on gelatin-coated glass slides and processed for Nissl staining.

#### Intracranial xenograft

Under isoflurane anesthesia, 6-week-old NOD-SCID mice were unilaterally injected in striatum with 3 × 10^5^ infected cells suspended in 3 μl of 1× PBS using the following coordinates from bregma: AP (Antero-Posterior) +0.5; ML (Medio-Lateral) +1.8; DV (Dorso-Ventral) −3.3 from the skull surface. Mice were euthanized upon observation of general condition or after 5 weeks from the injection; following anesthesia, mice were transcardially perfused with 4% PFA in PBS, and then brains were removed from the skull and kept in the same solution for O/N fixation. After fixation, brains were kept O/N in 30% sucrose in PBS and then embedded in O.C.T. for cryopreservation. The samples were cut coronally in 50-μm sections on cryostat (CM1850 UV, Leica). Subsequently, the sections were processed for immunofluorescence or mounted on gelatin-coated glass slides and processed for Nissl staining.

#### In vivo treatment

Orthotopic xenograft of CSCs was induced as described before. After 7 days, the mice, randomly divided in two groups, were injected at the same topological coordinates, with LV carrying either TetON-GFP or TetON-SES. A cohort of animals was euthanized after 28 days from the LV injection, and another cohort was left alive for perform survival rate and euthanized upon observation of general condition or at 90 days after the first surgery; following anesthesia, mice were transcardially perfused with 4% PFA in PBS, and then brains were removed from the skull and kept in the same solution for O/N fixation. After fixation, brains were kept O/N in 30% sucrose in PBS and then embedded in O.C.T. for cryopreservation. The samples were cut coronally in 50-μm sections on cryostat (CM1850 UV, Leica). Subsequently, the sections were processed for immunofluorescence or mounted on gelatin-coated glass slides and processed for Nissl staining.

#### Dox treatment

When appropriate, animals were treated with dox (60 mg/kg per mouse) through intraperitoneal injections with the following regimen: 3 days yes, 1 day no, 3 days yes, and then one injection every 2 days. No statistical methods were used to predetermine sample sizes. Mice were randomized before the procedures. Data collection and analyses were not performed blind to the conditions. Mice were maintained at the San Raffaele Scientific Institute Institutional Mouse Facility, and experiments were performed in accordance with experimental protocols approved by local Institutional Animal Care and Use Committees (#1051).

### Viral administration in wild-type mice

Striata of wild-type (WT) C57BL/6 animals were injected with LV (10^8^ IFU/ml) carrying either pEf1α-RFP or pMki67-GFP at the same coordinates previously used for the cell transplantation (AP +0.5; ML +1.8; DV −3.3 from the skull; 0.8 μl of an LV mixture). After 1 week from the surgery, the animals were euthanized for histological analyses. Hippocampi of WT C57BL/6 animals were injected with LV (10^8^ IFU/ml) carrying either GFP or SES (two injections per hippocampus AP −2.8; ML ±3; DV −3.5, −2.5; 0.8 μl each). After 1 month from the surgery, the animals were tested for behavioral tasks and euthanized for molecular and histological analyses.

### Immunostaining

Cells were seeded on glass coverslips (for CSCs previously coated with Matrigel to allow cell adhesion), and they were fixed for 20 min on ice in 4% PFA (Sigma-Aldrich) solution in PBS (Euroclone). Then, they were washed twice with PBS; were permeabilized for 30 min in blocking solution, containing 0.2% Triton X-100 (Sigma-Aldrich) and 5% donkey serum (Euroclone); and were incubated overnight at 4°C with the primary antibodies diluted in blocking solution. The next day, cells were washed three times with PBS for 5 min and incubated for 1 hour at room temperature with Hoechst 33342 (Thermo Fisher Scientific) and with secondary antibodies (Thermo Fisher Scientific) in blocking solution. Brain sections were blocked in 10% donkey serum and 0.2% Triton X-100 for 1 hour at room temperature. Incubation with primary antibodies was performed at 4°C O/N. Secondary antibodies were applied to sections for 2 hours at room temperature in blocking solution containing Hoechst 33342. Last, slices were washed and mounted in fluorescent mounting medium (Dako Cytomation). Images were acquired with an epifluorescence microscope Nikon DS-Qi2 and analyzed with Fiji software. The primary antibodies used were as follows: anti-V5 (1:500; mouse; Thermo Fisher Scientific, R96025), anti-GFP (1:1000; chicken; Thermo Fisher Scientific, A10262), anti-MAP2 (1:1000; chicken; Abcam, ab92434), anti–phospho-histone H3 (1:200; Ser^10^, rabbit; Sigma-Aldrich, 06-570), anti-CC3 (1:200; Asp^175^, rabbit; Cell Signaling Technology, 9661), anti-KI67 (1:500; clone SP6, rabbit; Immunological Sciences, MAB-90948), anti-human nuclei (1:500; mouse; Millipore, MAB1281), anti-human NESTIN (1:1000; mouse; Millipore, MAB5326), and anti-RFP (1:1000; rabbit; MBL, PM005). Antibodies used in this work are listed in table S9. For the immunofluorescence on cells, replicates showed in the bar graphs represent the number of coverslips from independent platings considered for each quantification. For the immunofluorescence on tissues, replicates showed in the bar graphs represent the number of sections.

### Nissl staining

Brain sections were rinsed in distilled H_2_O for 1 min and then stained in 0.1% cresyl violet solution boiled at 50°C for 7 min. Afterward, they were first rinsed in distilled H_2_O for 3 min and then washed in 70 to 100% ethanol serial dilutions for 1 min. Last, they were cleared in xylene for 2 hours and mounted with mounting solution (Eukitt, Sigma-Aldrich).

### MRI acquisition

MRI was performed on a small animal–dedicated 7T scanner (30/70 BioSpec; Bruker, Ettlingen, Germany). The animal protocol included high-resolution T2 sequence. Analysis of the tumor volume was performed using MIPAV (Medical Image Processing, Analysis, and Visualization) software (https://mipav.cit.nih.gov).

### Mouse behavioral testing

Animals were housed at a constant 23°C in a 12-hour light/12-hour dark cycle (lights off at 19:00), with food and water available ad libitum. We analyzed WT C57BL/6 mice, both males and females, at adult stage (ranging from 2 to 4 months of age) (all tests) infected 4 weeks before in their hippocampi with either GFP (mock) or SES. The sessions were recorded with the video tracking software EthoVision XT (Noldus). No statistical methods were used to predetermine sample sizes. Mice were randomized before the procedures. Data collection and analyses were not performed blind to the conditions.

#### Spontaneous alternation test

To test exploratory behavior and cognitive function related to spatial learning and memory, the mice were inserted in a four-arm maze and video-recorded for 10 min to evaluate the total number of the entries in all arms, the percentage of entries in each arm, and the consecution of the arm entries. This latter allows to identify pattern of behavior as follows (see also Fig. 7B): spontaneous alternation performance, a score index in which the visit of the four different arms without repetition is scored as 1, while at least one repetition in a string of four entrance is scored 0; alternate arm return, a score index in which at least one repetition in a string of three entrance is scored 1; and same arm return, a score index in which two consecutive entries in the same arm are scored 1.

#### Radial maze test

The eight-arm radial maze consisted of eight identical arms extending radially from an octagonal platform. It was elevated 80 cm above the floor and surrounded by external cues. A cup containing food was placed at the end of each arm. The protocol was divided into distinct phases: day 1, habituation at the apparatus for 10 min (without food at the end of the arms); day 2, food deprivation until when the animals had arrived at the 80 to 85% of their initial weigh, and during the experiment, the mice had to maintain this weight; day 3, training: put the food in half and at the end of each arm, release the mouse in the center of the arena, and it must eat two of eight pellets placed at the end of the arms; and days 4 to 13 (experimental days 1 to 10 in Fig. 7C), test: the pellets are placed only at the end of the eight arms. The mouse is released in the center of the arena to calculate (i) the time it takes to eat the eight pellets and (ii) the percentage of the incorrect choices (the mouse chose an empty arm) on the total entries. The maze was cleaned with water and 70% ethanol before the next mouse was placed on the apparatus.

#### Morris water maze test

The mice were inserted in a circular pool with a platform that allows them to escape the water (maximum length of each trial, 2 min). The release site can be in a different quadrant of the pool (see protocol in fig. S10F), with the position of the platform that was the same for the first 3 days and the reversal same for the last 2 days of the protocol. The time to complete each trial and the time spent in the platform zone and in the opposite quadrant were quantified.

### Human iPSC differentiation into neurons

WT iPSC cells were maintained in feeder-free conditions in mTeSR1 (STEMCELL Technologies) supplemented with Pen/Strept and seeded on human ESC–qualified Matrigel (Corning)–coated six-well plates; cells were fed daily and passaged in cell clumps weekly using Accutase solution (Sigma-Aldrich). At differentiation day −2, 90% confluent iPSC cultures were infected with the LV vector TetOn-Ngn2-T2A-Puro (*62*) in mTeSR1 medium supplemented with dox (2 μg/ml; Sigma-Aldrich) overnight. The next day, the medium was replaced with fresh mTeSR1 medium supplemented with antibiotic selection [puromycin (1 μg/ml), Sigma-Aldrich] and dox; dox was maintained for all the experiment. At day 0, the medium was replaced with differentiation medium “mTeSR1 + LSBX.” The differentiation medium was replaced daily according to the following scheme: days 0 and 1: mTeSR1 + LSBX; days 2 and 3: mTeSR1 + LSBX + PSD; days 4 and 5: two-thirds mTeSR1 + one-third N-2 medium + LSX + PSD; and days 6 and 7: one-third mTeSR1 + two-thirds N-2 medium + PSD. At day 8, cells were detached by Accutase solution incubation at 37°C for 20 min to obtain a single-cell suspension. Cells were centrifuged, counted, and seeded at a density of 55,000 cells/cm^2^ onto poly-l-lysine/laminin/fibronectin–coated plates or coverslip in neuronal maturation medium supplemented with ROCK inhibitor Y27632 (10 μM; Selleckchem) for the first 24 hours. The culture medium was replaced the next day to remove the ROCK inhibitor, and then half of the medium was replaced with fresh neuronal maturation medium twice a week. LSBX: LDN193189 (250 nm; Stemgent), SB431542 (10 μM; Sigma-Aldrich), and XAV939 (5 μM; Sigma-Aldrich). PSD: PD0325901 (8 μM; Sigma-Aldrich), SU5402 (10 μM; Sigma-Aldrich), and DAPT (10 μM; Sigma-Aldrich). N-2 medium: DMEM/F12 with B-27 supplement (0.5×; Thermo Fisher Scientific) and N-2 supplement (0.5×; Thermo Fisher Scientific). Neuronal maturation medium: Neurobasal A (Thermo Fisher Scientific) supplemented with 1× B-27 supplement, 2 mM glutamine, 1% Pen/Strept, brain-derived neurotrophic factor (20 ng/ml; PeproTech), ascorbic acid (100 nM; Sigma-Aldrich), laminin (1 μg/μl), DAPT (10 μM), and dibutyryl cyclic adenosine monophosphate (250 μM; Selleckchem).

### Primary mouse neuronal cultures

Primary cultures of mouse embryonic cortical neurons were prepared from E17.5 (embryonic day 17.5) C57BL/6 WT mice. Briefly, after dissection, cortices were enzymatically digested with 0.025% trypsin (Gibco) in Hank’s balanced salt solution (HBSS) (Euroclone) for 20 min at 37°C. Successively, HBSS with trypsin was removed, and the hippocampi were washed with plating medium (Neurobasal A medium supplemented with 1× B-27 supplement, 3.3 mM glucose, 2 mM glutamine, and 1% Pen/Strept) and mechanically dissociated using a P1000 pipette to obtain a homogeneous cell suspension. Cells were then plated on poly-l-lysine–coated (0.1 mg/ml) glass coverslips.

## Notes

View/request a protocol for this paper from *Bio-protocol*.
