# Supplementary material for: A SOX2-engineered epigenetic silencer factor represses the glioblastoma genetic program and restrains tumor development
Source: Sci Adv. 2022 Aug 3;8(31):eabn3986. doi: 10.1126/sciadv.abn3986 (PMC9348799; doi:10.1126/sciadv.abn3986)
Supplement: Supplementary file 1 — Figs. S1 to S12 [file sciadv.abn3986_sm.pdf]

Science Advances
AAAS

Supplementary Materials for

A SOX2-engineered epigenetic silencer factor represses the glioblastoma genetic program and restrains tumor development

Valerio Benedetti *et al.*

Corresponding author: Vania Broccoli, broccoli.vania@hsr.it; Alessandro Sessa, sessa.alessandro@hsr.it

Sci. Adv. **8**, eabn3986 (2022)
DOI: 10.1126/sciadv.abn3986

The PDF file includes:

Figs. S1 to S12
Legends for tables S1 to S10

Other Supplementary Material for this manuscript includes the following:

Tables S1 to S10

Benedetti, Banfi et al.,

Supplementary Figures

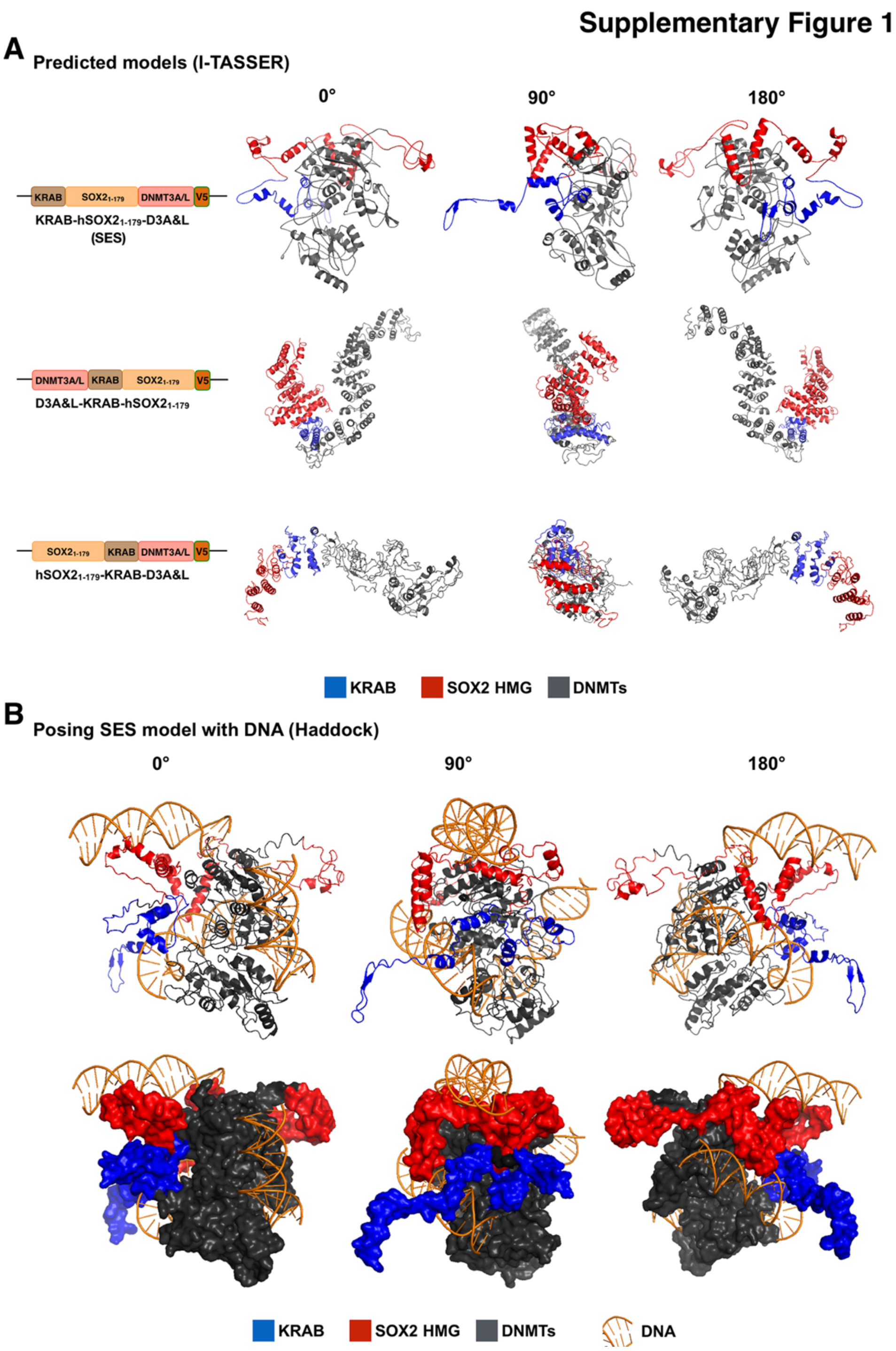

Fig. S1. SES *in silico* modeling. (**A**) *In silico* predicted structure of the artificial factors (based on three different configurations of human SOX2 transcription factor and the epigenetic domain KRAB, DNMT3a and DNMT3L as indicated on the left) obtained with I-TASSER and shown in three different angles as indicated. The SOX2 HMG box domain is highlighted in red, while KRAB domain and DNMTs are in blue and gray, respectively. SES protein shows the best folding among the models generated (see also **Table S1**). (**B**) Docking simulation between the SES protein model (up: cartoon,

bottom: surface) and 19 DNA nucleotides. The top 3 poses of docking were filtered by **Δ**G of binding of -300Kcal/mol. Here the best pose for each of these three putative docking sites on SES is shown. This analysis supports the possibility for SES to bind DNA in three different positions including the HMG box domain with the DNMT domains in its proximity.

Supplementary Figure 2

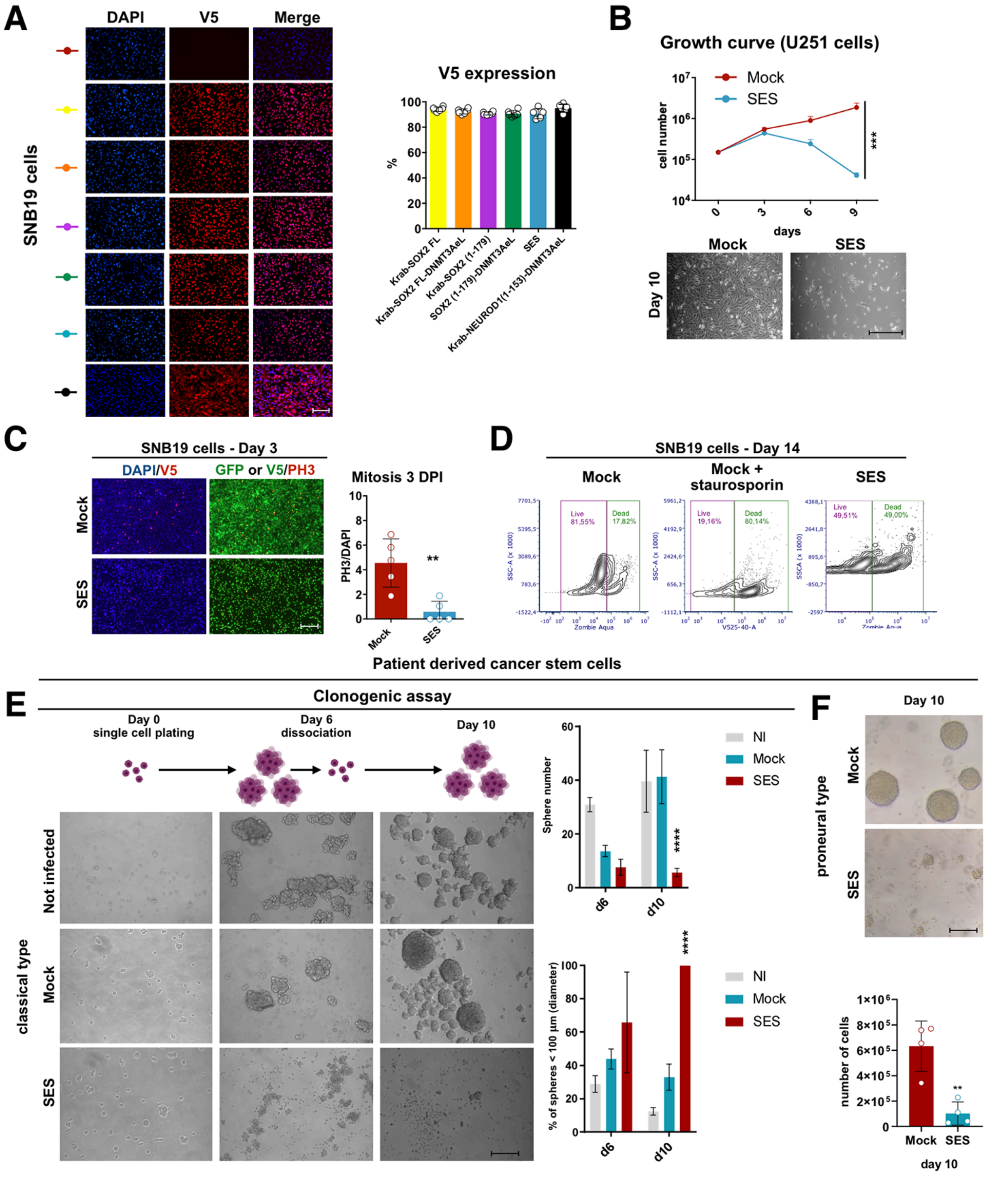

Fig. S2. SES activity *in vitro*. (**A**) Infection efficiency (mean +/- SEM) of the different lentiviruses indicated Fig. 1A in SNB19 cells. *n*=6. (**B**) Up, growth curve of U251 cells, ***p = 0.0005; statistically compared with two-way ANOVA(*n*=3); bottom, microphotographs of the U251 cells after 10 days from the infection with either mock (GFP) or SES. (**C**) Left, immunocytochemistry for PH3 mitotic marker and for V5 tag, counterstained with DAPI in SNB19 cells 3 days after GFP or SES infection. Quantification as percentage of $PH3^+$ cells on the total number of DAPI nuclei (mean

+/- SEM); **p = 0.0033; statistically compared using unpaired t test. *n*=5. (**D**) Fluorescent Activated Cell Sorting analysis using the dead cell-penetrant Zombie Aqua dye in both mock and SES infected SNB19 cells after 14 days from transduction. Mock cells treated with 2 µm Staurosporin (12 hrs) was added as positive control. Gating strategy was based on unstained cells. (**E**) CSCs (classical type) either not infected or infected with the lentiviruses carrying GFP or SES are assayed for their clonogenic potential using sphere number and size (percentage of spheres below 100um in diameter) at the indicated time points as parameters (mean +/- SEM). ****p < 0.0001; statistically compared with two-way ANOVA. *n*=3. (**F**) Microphotographs and count of cell number after 10 days from the Mock/SES infection of CSCs (proneural type) in floating culture (mean +/- SEM). **p = 0.0029; statistically compared with unpaired *t* test. *n*=4. Bars: A: 100 µm; B, C: 200 µm. E, F: 300 µm.

Supplementary Figure 3

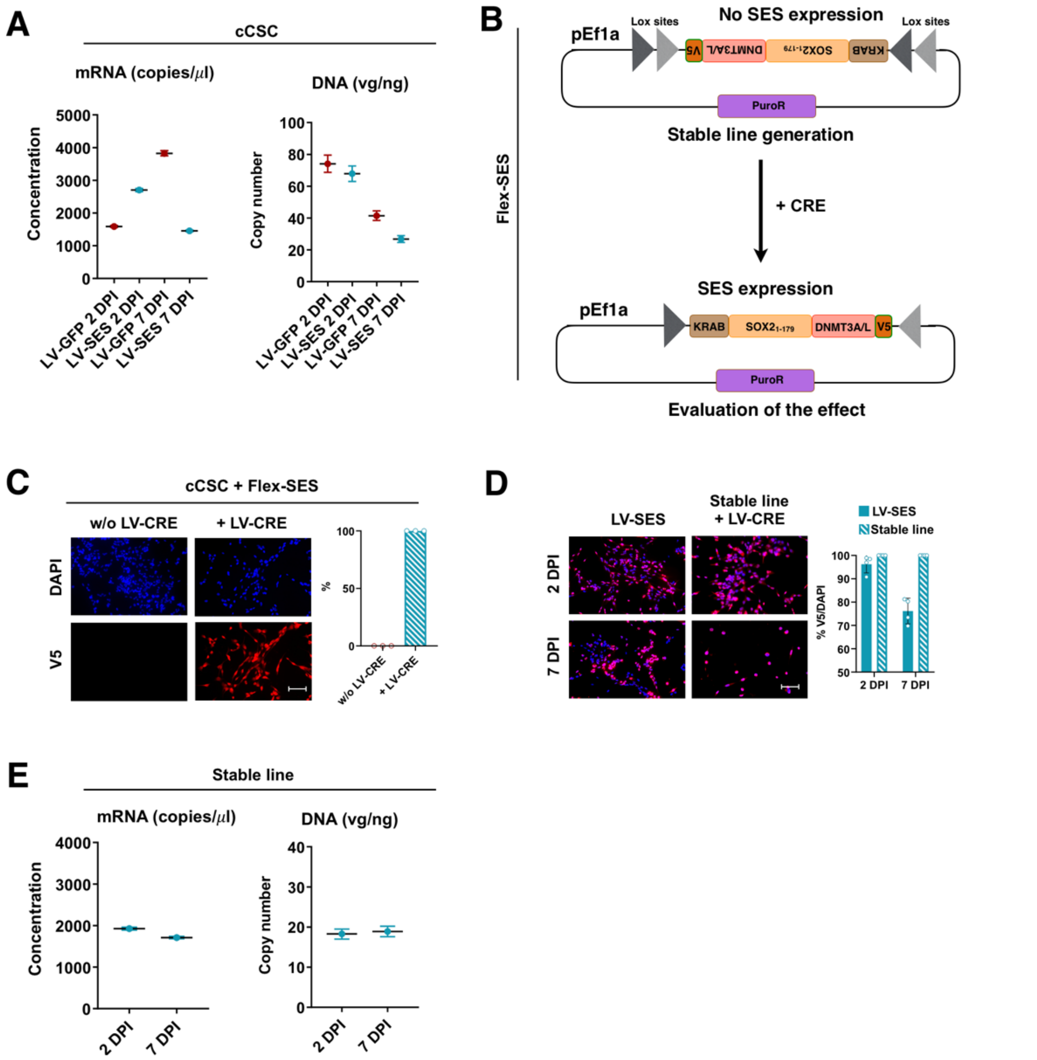

Fig. S3. SES long expression *in vitro*. (**A**) Graphs showing RNA concentration (for cDNA expressed as copies of detected molecules/µl) and viral genome (vg) copy number (for gDNA) in classical CSCs infected with either LV-GFP or LV-SES at 2 and 7 DPI quantified by digital droplet PCR. The error bars show the 95 % confidence intervals of the Poisson distribution. (**B**) Schematic representation of Flex system for SES: SES expression is allowed only after Cre recombination. Puromycin resistance (PuroR) grants the possibility to obtain stable line with construct integration. (**C**) Flex-SES used in CSCs shows that all the cells are positive for V5 tag when Cre is administered (by LV) (after Puro selection). *n*=3. (**D**) Comparison of the V5^{+} cells either 2 or 7 days post infection (DPI) in both CSCs + LV-SES and CSCs stable line + LV-CRE. *n*=4. (**E**) Graphs showing RNA concentration (for cDNA expressed as copies of detected molecules/µl) and viral genome (vg) copy number (for gDNA) in CSCs stable line + LV-CRE at 2 and 7 DPI quantified by digital droplet PCR. The error bars show the 95 % confidence intervals of the Poisson distribution.

Supplementary Figure 4

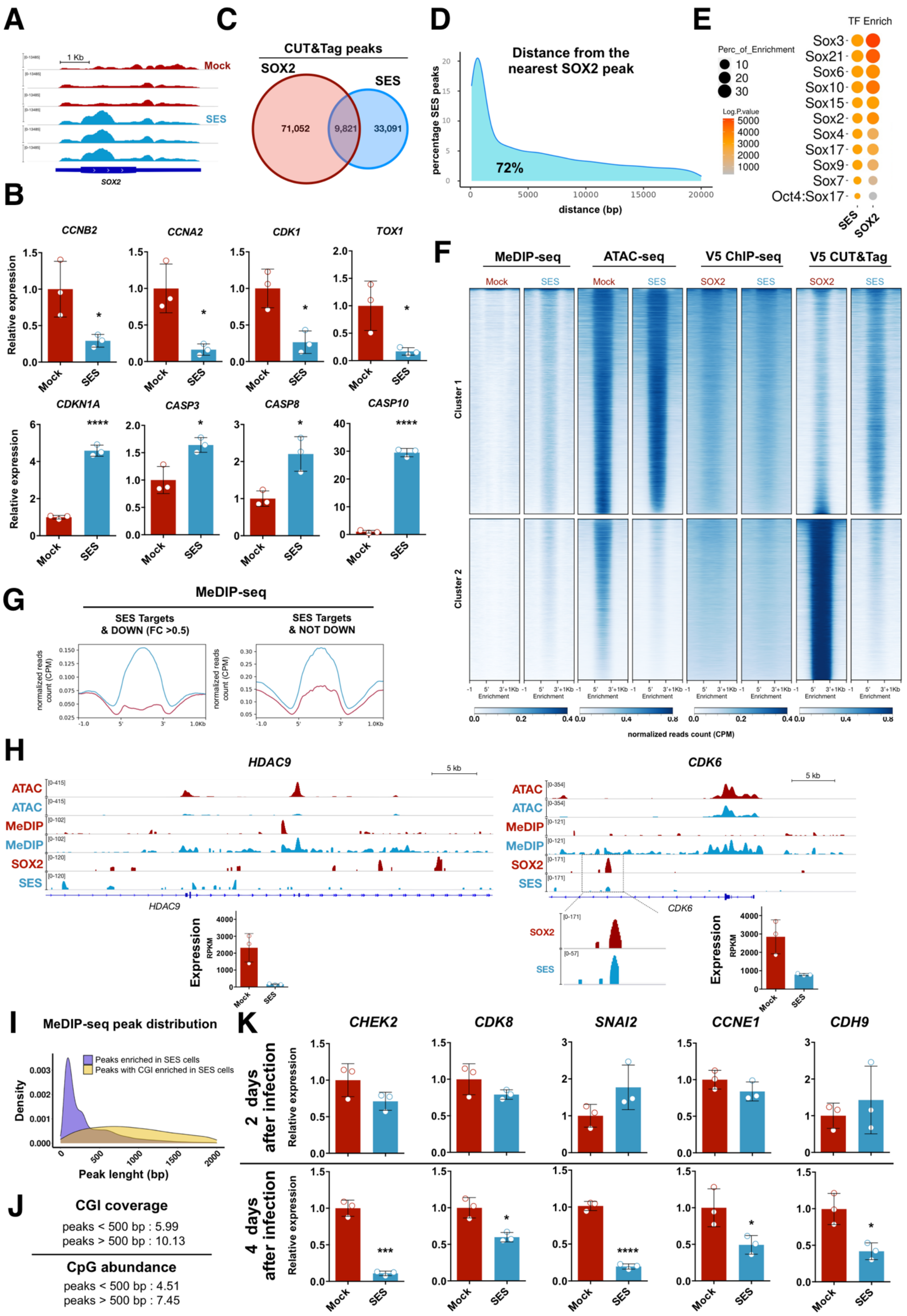

J

CGI coverage

peaks < 500 bp : 5.99
peaks > 500 bp : 10.13

CpG abundance

peaks < 500 bp : 4.51
peaks > 500 bp : 7.45

Fig. S4. SES transcriptional and epigenetic activities. (**A**) IGV snapshot of SOX2 locus showing RNA-seq tracks in both mock (red tracks) and SES (blue tracks) infected cells evidenced the overexpression of SES RNA that share with *SOX2* its 5' part. (**B**) RT-qPCR validation of deregulated genes identify by RNA-seq (mean +/- SEM). *CCNB2*: * p = 0.0352; *CCNA2*: * p = 0.0132; *CDK1*: * p = 0.0141; *TOX1*: * p = 0.0342; *CCNB2*: **** p < 0.0001; *CASP3*: * p = 0.0168; *CASP8*: * p = 0.0151; *CASP10*: **** p < 0.0001; statistically compared with unpaired t test. *n*=3. (**C**) Venn Diagram showing the overlap between SOX2 and SES peaks identified by CUT&Tag experiment. (**D**) SES peak distribution, as distance (in base pairs, bp) from the nearest SOX2, of the 72% SES peaks that fall within 20kb from SOX2 peaks. (**E**) Evaluation of the enrichment of transcription factor binding sites of the SOX family in both SOX2 and SES peaks using HOMER (Hypergeometric Optimization of Motif EnRichment) analysis (see **Table S4**). **(F)** Heat-maps showing relative enrichment for MeDIP-seq, ATAC-seq, ChIPseq (V5 tag for SOX2 and SES overexpression) and CUT&Tag (V5 tag for SOX2 and SES overexpression) in both control and SES condition in cluster 1 of the heat-map of the **Fig. 2D** (n = 63,749 unique rows). Enrichments are showed as color scale in peaks bodies -/+ 1 Kb. (**G**) Density plots summarizing the mean of the MeDIP signals of SES targets either downregulated (left) or not downregulated (right). (**H**) IGV snapshots of SOX2 targets showing both ATAC-seq, MeDIP-seq and V5-ChIP-seq tracks in both mock (red tracks) and SES (blue tracks) infected cells indicating strong chromatin remodeling around the SOX2/SES binding sites. Expression data (RPKM from RNA-seq experiments) are also shown on the bottom. (**I**) Distribution of peak length of the MeDIP-seq peaks enriched in SES infected cells compared to the mock (violet) and the fraction of the same peaks that contain at least one CGI. (**J**) Up, percentage of the DNA regions underlying the peaks that falls in CGIs in MeDIP-seq peaks enriched in SES infected cells compared to the mock, divided in short (< 500 bp) and long (> 500 bp). Below, percentage of CpG di-nucleotide in MeDIP-seq peaks enriched in SES infected cells compared to the mock, divided in short (< 500 bp) and long (> 500 bp). (**K**) RT-qPCR on SES target genes at 2 and 4 days after mock (GFP)/SES infection indicating that certain loci may need prolonged SES expression to be downregulated (mean +/- SEM). *CHEK2 2dd*: p = 0.1236; *CHECK2 4dd*: *** p = 0.0002; *CDK8 2dd*: p = 0.1767; *CDK8 4dd*: * p = 0.0104; *SNAI2 2dd*: p = 0.1206; *SNAI2 4dd*: **** p < 0.0001; *CCNE1 2dd*: p = 0.2021; *CCNE1 4dd*: * p = 0.0384; *CDH9 2dd*: p = 0.4931; *CDH9 4dd*: * p = 0.0145; statistically compared with unpaired t test. *n*=3.

Supplementary Figure 5

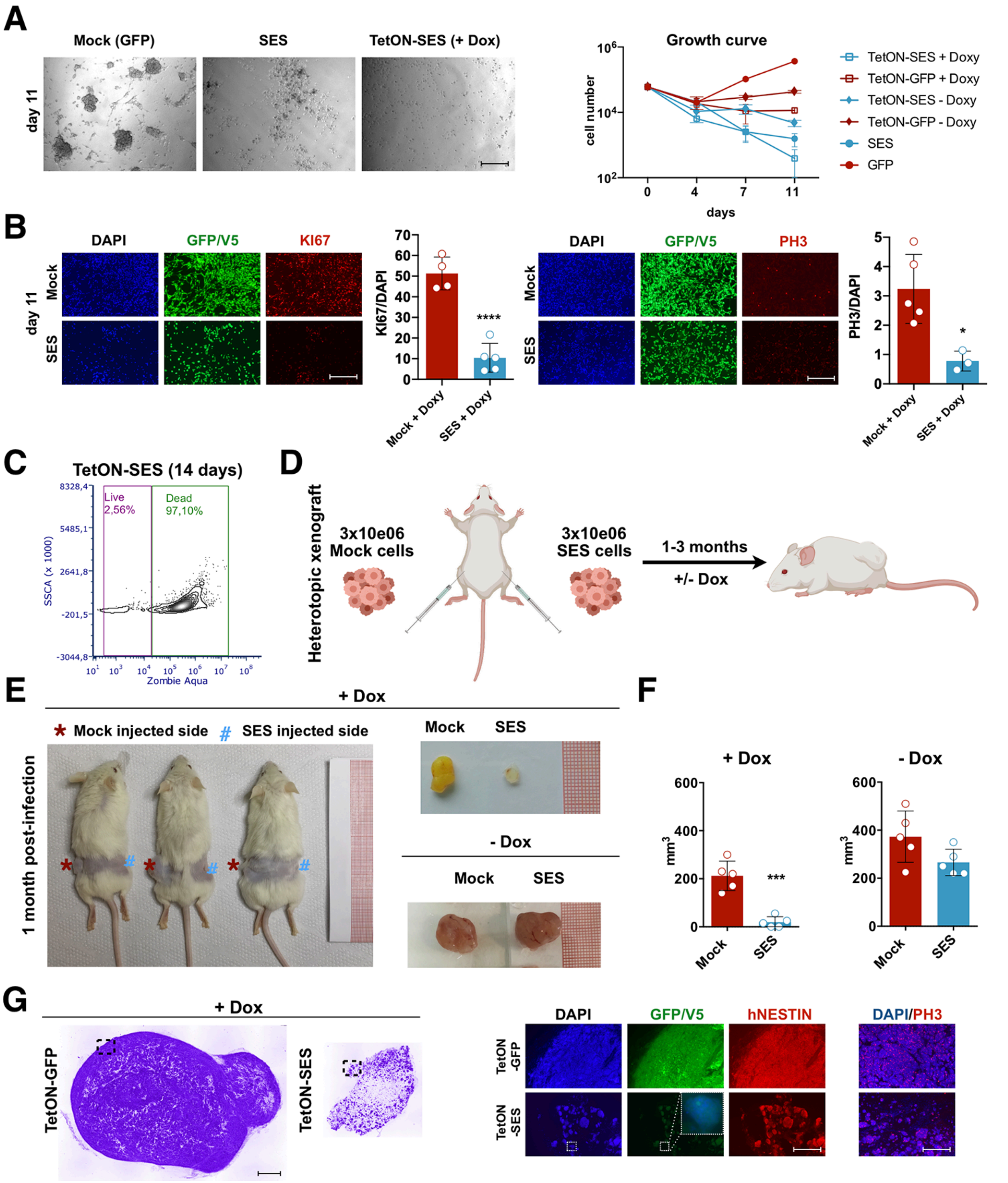

Fig. S5. SES antitumor activity in cancer cells and subcutaneous xenografts. (A) Microphotographs and growth curves of patient-derived cancer stem cells (CSCs) of the classical GBM subtype transduced with the indicated constructs including TetOn-GFP and TetOn-SES demonstrated the efficacy of the latter when dox is applied. Dox itself displays a detrimental effect for CSC growth. Growth curve: GFP vs TetON-SES (+Dox): ****$p < 0.0001$; TetON-GFP vs TetON-SES (+Dox): *$p = 0.0288$; GFP vs TetON-GFP (+Dox): ****$p < 0.0001$; TetON-SES (-Dox) vs TetON-SES (+Dox): *$p = 0.0235$; GFP vs TetON-SES (-Dox) ****$p < 0.0001$; GFP vs TetON-

GFP (+Dox) ****p < 0.0001; TetON-GFP (-Dox) vs TetON-GFP (+Dox): *p = 0.0135; statistically compared with two-way ANOVA (n=3). (**B**) Left, immunocytochemistry for KI67, GFP and V5 tag, counterstained with DAPI in CSCs 11 days after GFP or SES infection. Quantification as percentage of $KI67^+$ cells on the total number of DAPI nuclei (mean +/- SEM); p < 0.0001; statistically compared using unpaired *t* test. n=4-5. Right, immunocytochemistry for PH3, GFP and V5 tag, counterstained with DAPI in CSCs 4 days after TetOn-GFP or TetOn-SES infection with Dox. Quantification as percentage of $PH3^+$ cells on the total number of DAPI nuclei (mean +/- SEM); p = 0.0138; statistically compared using unpaired *t* test. *n*=3-5. (**C**) Fluorescent Activated Cell Sorting analysis using the dead cell-penetrant Zombie Aqua dye in TetOn-SES (+dox) infected CSCs after 14 days from transduction. Gating strategy is based on unstained cells. (**D**) Schematic representation of the subcutaneous heterotopic xenografts with 3x10e6 CSCs (classical type, pre-infected with either mock (Tta and TetOn-GFP) or (Tta and TetOn-SES) in NSG mice (+/- dox). (**E**) Left, photograph of animals injected and treated with dox after the sacrifice (one month after the injection), red asterisk = Mock cells injected side, blue pound = SES cells injected sides. Right: photographs of the resulting subcutaneous tumors. (**F**) Quantification of tumor volume (mean +/- SEM): + Dox: *** p = 0.0002; - Dox: p = 0.019. Statistically compared with unpaired *t* test. *n*=5 from 5 animals. (**G**) Left, Nissl staining of tumors found in mock injected and SES injected sites in dox treated animals. Right, immunofluorescence on the same tumors indicates that mock tumors are formed by GFP^+ $hNESTIN^+$ cells while very few $V5^+$ $hNESTIN^+$ cells have been detected in SES treated tumors (composed by $hNESTIN^+$ cells). Both tumors contain proliferative $PH3^+$ cells. Bars: A: 300 µm; B = 200 µm G left: 1 mm; Gright: 200 µm.

Supplementary Figure 6

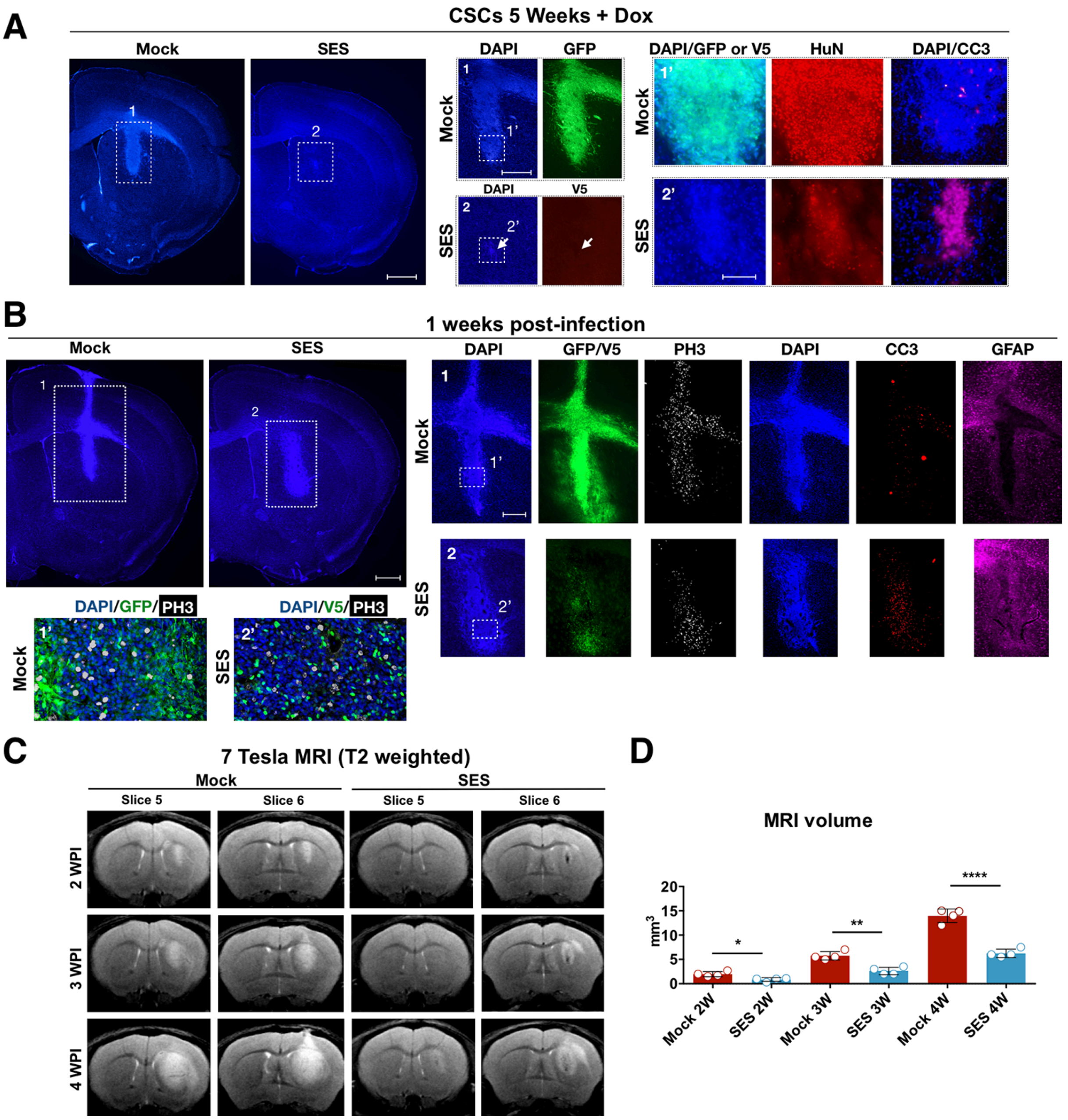

Fig. S6. Tet-On SES antitumor activity *in vivo*. (**A**) Brain coronal sections of animals transplanted with mock or SES infected CSCs stained for GFP/V5 and human nuclei (HuN) and for cleaved Caspase-3 (CC3) counterstained with DAPI. (**B**) Brain coronal sections stained for GFP/V5 and PH3 (middle) and cleaved Caspase-3 (CC3) and GFAP (right) from animals that received naive CSCs followed by stereotactic injections of mock and SES expressing lentiviruses 1 week later (representative of 2 animals/group). (**C**) Examples of MRI scanning at 2, 3 and 4 weeks post-infection (WPI) (two slices for both one mock (+dox) and one SES (+dox) treated mouse). (**D**) Tumor volume as measured by hyper-intensity of T2-weighted imaging (MIPAV software) (mean +/- SEM). 2

Weeks: * $p = 0.0128$; 3 Weeks: ** $p = 0.0016$; 3 Weeks: ****$p < 0.0001$; statistically compared with unpaired *t* test. *n*=5 from 5 animals. Bars: A left and B left: 1 mm; A1 and B1: 300 µm; A2': 50 µm.

Supplementary Figure 7

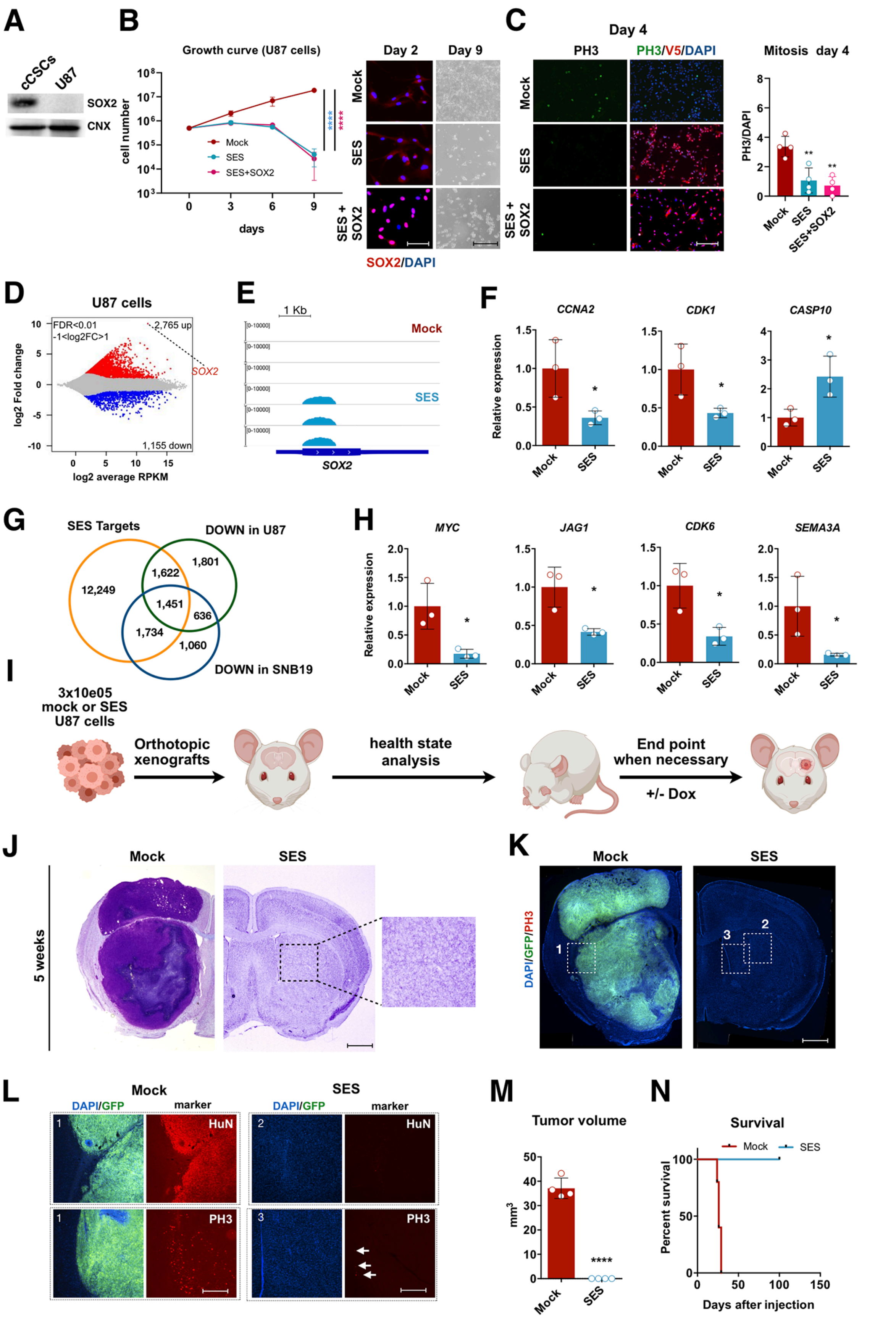

Fig. S7. SES expression erases the tumorigenicity of Sox2$^-$ U87 glioma cells. (**A**) Western blot (WB) for SOX2 and Calnexin (CNX) (as loading control) in U87 cells and human classic CSCs

confirms that U87 are SOX2$^-$ glioma cancer cell line. (**B**) Left, growth curve of U87 cells infected with either mock (GFP), SES alone or SES + full length SOX2, ****p < 0.0001; statistically compared with two-way ANOVA (n=3). Middle, Immunofluorescence of the cells for SOX2 counterstained with DAPI after 2 days from the infection. Right, microphotographs of the cells after 9 days from the infection. (**C**) Immunofluorescence for mitosis marker PH3 and V5 tag in both mock, SES and SES+SOX2 transduced U87 cells (4 days after infection). Quantification (mean +/- SEM): SES **p = 0.0040, SES+SOX2 **p = 0.0016. Statistically compared with one-way ANOVA. *n*=4. (**D**) SES causes massive gene deregulation in U87 cells already 2 days the infection, as assessed by RNA-seq. (**E**) IGV snapshot of the SOX2 locus, showing RNA-seq tracks in both mock (red tracks) and SES (blue tracks) infected cells, evidences the overexpression of SES RNA that share with *SOX2* the 5' part. (**F**) Validation of deregulated genes as resulted in RNA-seq datasets, by RT-qPCR (mean +/- SEM). *CCNA2*: * p = 0.0445; *CDK1*: * p = 0.0436; *CASP10*: * p = 0.0326; statistically compared with unpaired t test. *n*=3. (**G**) Venn Diagram showing the overlap between SES target genes (orange circle), and genes downregulated (FDR < 0.1, fold change < 0) in U87 (green circle) and in SNB19 (blue circle). (**H**) Validation of downregulated SOX2 target genes as resulted in RNA-seq datasets, by RT-qPCR (mean +/- SEM). *MYC*: * p = 0.0242; *JAG1*: * p = 0.0186; *CDK6*: * p = 0.0216; *SEMA3A*: * p = 0.0490; statistically compared with unpaired t test. *n*=3. (**I**) Illustration of the analysis performed in NSG mice transplanted with U87 cells pre-infected with either mock (GFP) or SES expressing viruses. (**J**) Nissl staining of representative brain sections of animals transplanted with either GFP or SES U87 cells (1 month after the graft) indicating that mock U87 cells always generated massive GFP$^+$ tumors able to invade also the cortex, while no tumors are found in brains transplanted with SES$^+$ U87 cells. (**K**) Immunohistochemistry for GFP and V5 counterstained with to visualize remaining transduced CSCs in both conditions. We never detected any V5$^+$ cells in SES samples. (**L**) Tumors originating from mock cells are formed by human nuclei (HuN) positive, PH3$^+$ proliferating cells, while virtually no human cells are retrieved in brain injected with SES expressing cells. Of note, the few PH3$^+$ cells present (white arrows), are located close to the lateral ventricles, being presumably mouse neural precursors in active cell division. (**M**) Quantification of tumor volume (mean +/- SEM): **** p < 0.0000; - Dox: p = 0.7731. Statistically compared with unpaired t test. *n*=4 from 4 tumors. (**N**) Kaplan-Meier curve shows that mice injected with mock cells died within a month from the surgery while SES receiving animals remain in good health and the time of sacrifice (100 days after the transplant) (n = 5 animals per group). Bars: B right: 100 μm; B right: 200 μm; C: 100 μm; J, K: 1 mm; L: 200 μm

Supplementary Figure 8

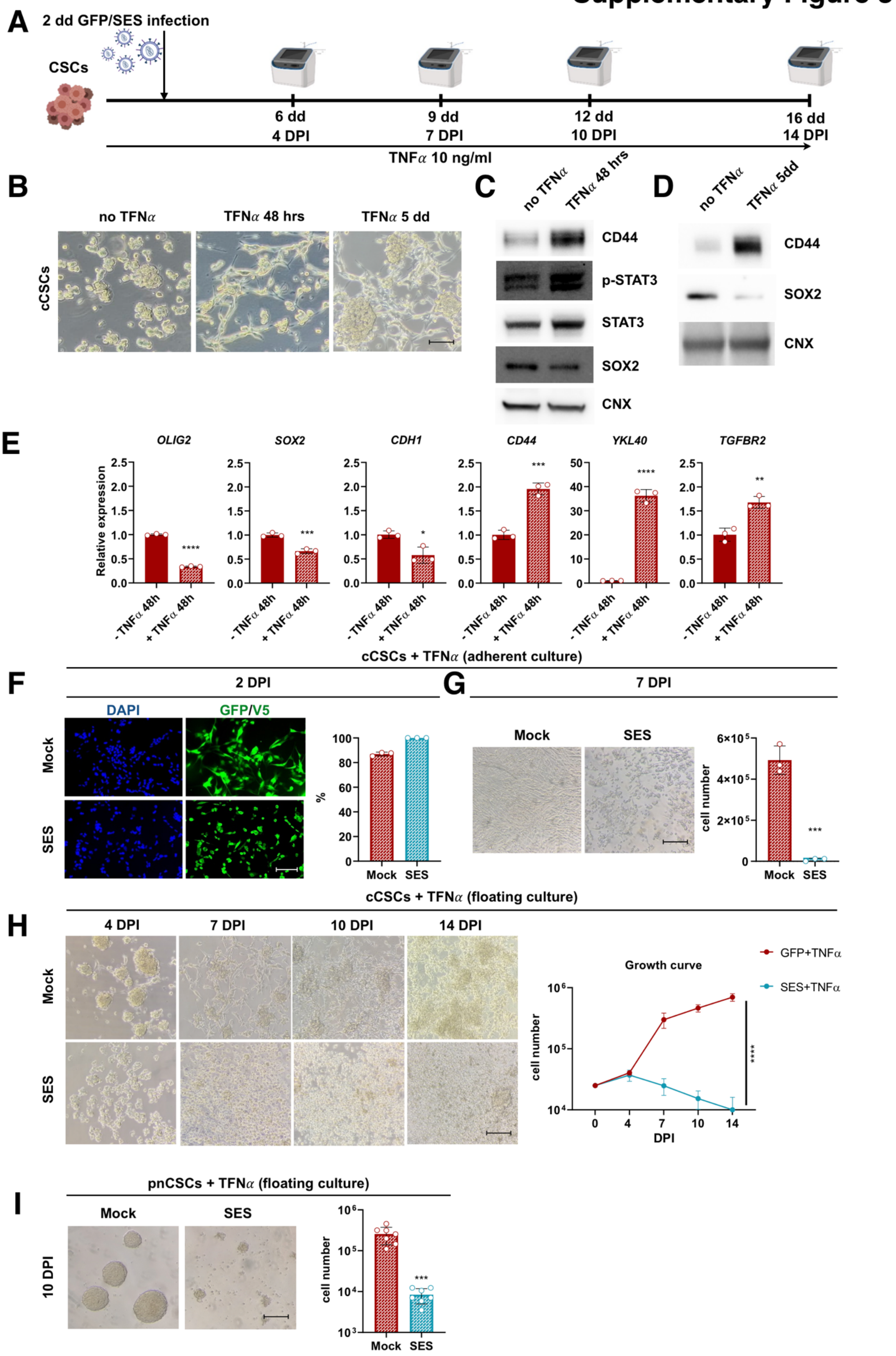

Fig. S8. SES is active in TNFα-induced mesenchymal CSCs. (**A**) Schematic representation of the experimental mesenchymal shift (cCSCs to mCSCs) due to TNFα: cCSC are continuously treated with TNFα (10 ng/ml), and infected (GFP or SES) 2 days after the treatment start. Cell growth is evaluated at 4, 7, 10 and 14 days post infection (DPI). (**B**) Appearance of the cells untreated or after 2 days and 5 days of TNFα. (**C**) WB for mesenchymal markers CD44 and p-STAT3/STAT3, SOX2 and calnexin (as loading control) confirms the mesenchymal shift after 2 days of TNFα. (**D**) WB for mesenchymal marker CD44, SOX2 and calnexin (as loading control) indicates the strong SOX2 inhibition after 5 days of TNFα. (**E**) RT-qPCR analysis of deregulated genes after 2 days of TNFα (mean +/- SEM). *OLIG2*: **** $p < 0.0001$; *SOX2*: *** $p = 0.0009$; *CDH1*: *** $p = 0.0142$; *CD44*: *** $p = 0.0005$; *YKL40*: **** $p < 0.0001$; *TGFBR2*: ** $p = 0.0032$; statistically compared with unpaired *t* test. n=3. (**F**) Immunocytochemistry for GFP and V5 tag, counterstained with DAPI in CSCs after 4 days of TNFα and 2 DPI. Quantification as percentage of GFP^+ or $V5^+$ cells on the total number of DAPI nuclei (mean +/- SEM). n=3. (**G**) Microphotographs of CSCs in adherent culture after 9 days of TNFα (7 DPI). Quantification as number of live cells in either not GFP infected (Mock) or SES infected (mean +/- SEM): *** $p = 0.0003$; statistically compared with unpaired *t* test. n=3. (**H**) Microphotographs and growth curve of CSCs in floating culture along TNFα treatment 4, 7, 10 and 14 DPI. Growth curve: **** $p < 0.0001$; statistically compared with two-way ANOVA. n=3. (**I**) Microphotographs and count of CSCs (proneural type) in floating culture along TNFα treatment 10 DPI, (mean +/- SEM): ***$p = 0.0001$ statistically compared with unpaired *t* test. n=7. Bars: B, F: 100 μm; G: 200 μm; I: 400 μm.

Supplementary Figure 9

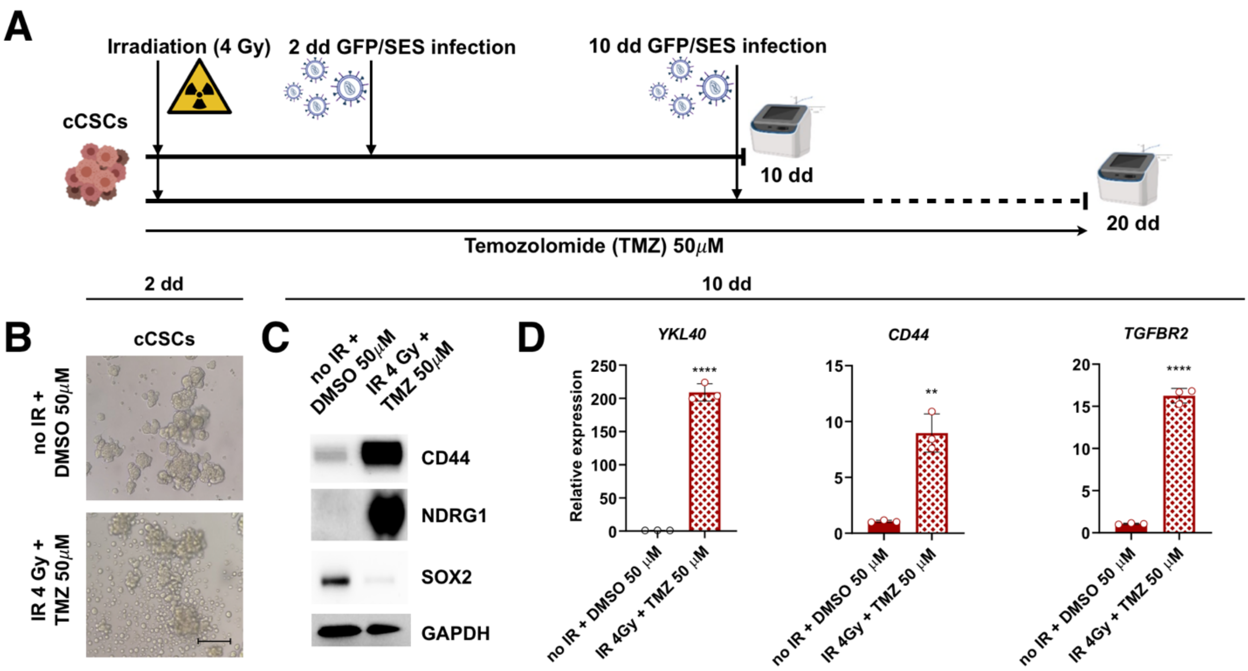

Fig. S9 Treatment-induced mesenchymal shift in CSCs. (**A**) Schematic representation of the experimental mesenchymal shift (cCSCs to mCSCs) due to standard GBM care and SES treatment: cCSC are continuously treated with Temozolomide (TMZ, 50 µM) , irradiated (IR, 4 Gy) and infected (GFP or SES) either 2 or 10 days after. Cell growth is then evaluated at 10 and 20 days from the start of the procedure, respectively. (**B**) Appearance of the cells after 2 days of IR+TMZ (or no IR + DMSO as control). (**C**) WB for mesenchymal markers CD44 and NDRG1, SOX2 and GAPDH (as loading control) confirms the mesenchymal shift after 10 days of IR+TMZ. (**D**) RT-qPCR analysis of deregulated genes after 10 days of IR+TMZ (mean +/- SEM). *YKL40*: **** $p < 0.0001$; *CD44*: ** $p = 0.0013$; *TGFBR2*: **** $p < 0.0001$; statistically compared with unpaired *t* test. *n*=3. Bar: B: 100 µm.

Supplementary Figure 10

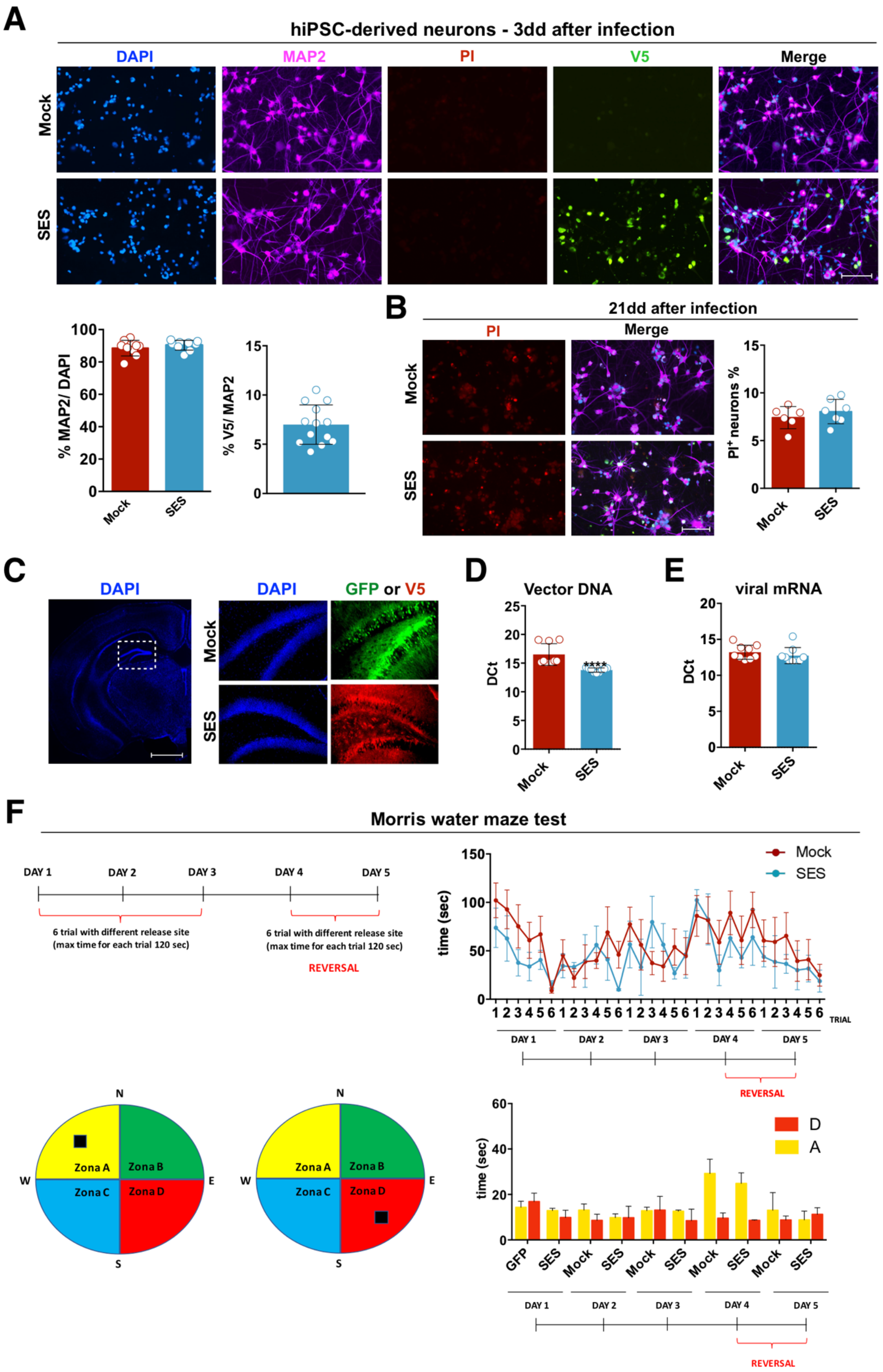

Fig. S10. SES expression in the adult mouse brain. (**A, B**) Analysis of cell viability of human iPSC-derived neuronal cultures infected with either GFP (mock) or SES expressing viruses by staining for

PI, V5 and MAP2 at 3 (**A**) and 21 (**B**) days post-transduction. Quantification (mean +/- SEM) 3 days after infection: MAP2/DAPI p = 0. 3616 (n=9). Quantification (mean +/- SEM) 21 days after infection: p = 0.3790 (n=6). (**C**) Hippocampal coronal sections stained for GFP, V5 and DAPI confirming the extensive expression of either GFP or SES-V5. (**D**) Viral genome quantification shown as Delta Ct between viral genome and nuclear DNA (mean +/- SEM): *** p = 0.0006 (n=3). (**E**) Quantification of viral mRNA (RT-qPCR) shown as Delta Ct between viral mRNA and 18S of the host cells (mean +/- SEM): p = 0.3603 (n=3). (**F**) Morris water maze test results. On the left, the protocol used with repetitive trials and different release sites. On the right, both the graph of the time used to accomplish the task (up) and the quantification of the time spent in a defined platform area (yellow) or in the opposite one (red) indicate no differences between the two mouse groups. Time for the task: p = 0.2406. Statistically compared with two-way ANOVA. Time in the A/D areas: p > 0.9999. Statistically compared with Mann-Whitney test. n=5 animals per group. Bars: A, B: 200 μm; C: 1 mm.

Supplementary Figure 11

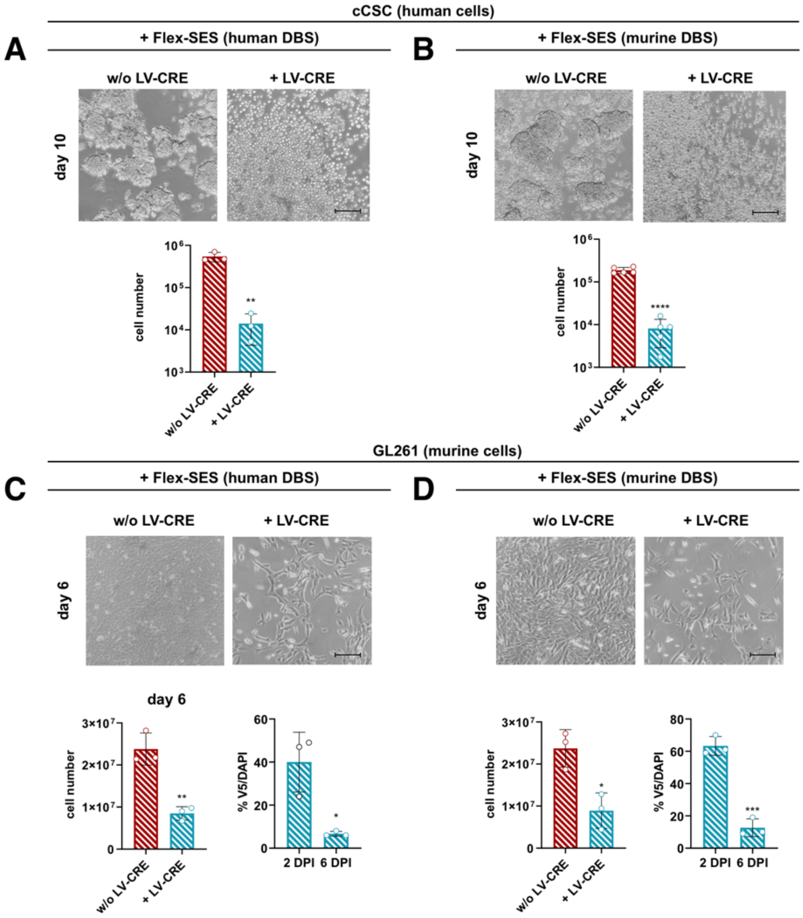

Fig. S11. *In vitro* comparison between human and murine SES. (**A-B**) Microphotographs and count of stable line of human CSCs (classical type) established with Flex-SES with either human (**A**) or murine (**B**) SOX2 domain, with or without LV-CRE (after 10 days of infection), (mean +/- SEM): human **p = 0.0025 (n=3); murine **p < 0.0001 (n=4); statistically compared with unpaired *t* test. (**C-D**) Microphotographs and count of stable line of murine glioma line GL261 as stable line established with Flex-SES with either human (**C**) or murine (**D**) SOX2 domain, with or without LV-CRE (after 6 days of infection), (mean +/- SEM): human **p = 0.0031 (n=3); murine *p = 0.0144 (n=3); statistically compared with unpaired *t* test. Quantification of $V5^+$ cells on the total is also shown, (mean +/- SEM): human *p = 0.0140 (n=3); murine ***p = 0.0004 (n=3); statistically compared with unpaired *t* test. Bars: A, B: 500 µm; C, D: 200 µm.

Supplementary Figure 12

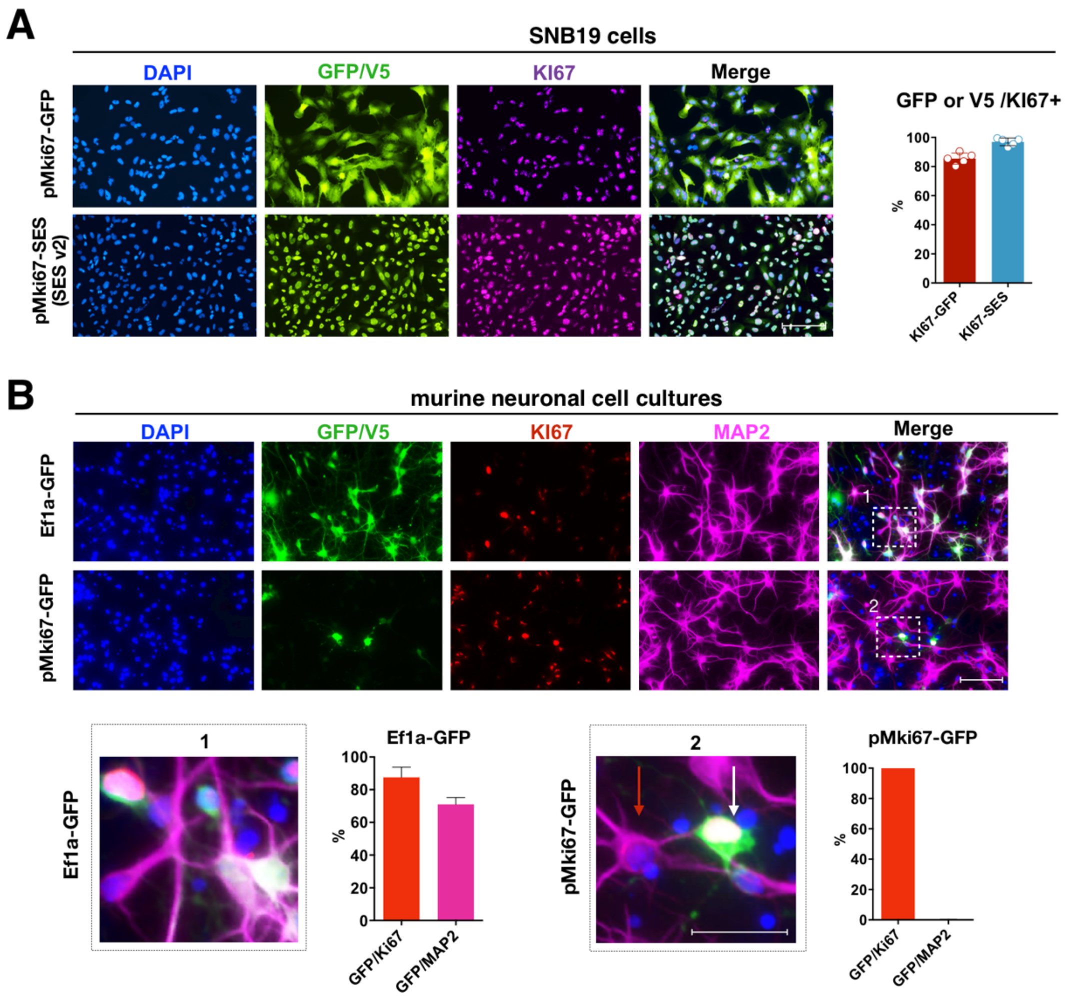

Fig. S12. ***In vitro*** **functional assessment of the Mki67 promoter.** (**A**) GFP, V5, KI67 (mitotic marker) immunostainings in SNB19 cells infected with either GFP (upper part) or SES (bottom part) under the control of the Mki67 promoter. The Mki67 promoter efficiently drives transgene expression in KI67^{+} proliferating cells. Quantitative analysis on the right (mean +/- SEM). n=5 (**B**) Immunofluorescence for GFP, V5, KI67 (mitotic marker) and MAP2 (neuronal marker) on mouse primary cortical cultures shows that GFP when driven by the pMki67 is detected only in KI67^{+} glial proliferating cells (i.e. white arrow in inset 2), but never in MAP2^{+} neurons (red arrow in inset 2). Conversely, constitutive GFP is found in virtually all cells as expected. Quantifications are provided (mean +/- SEM). n=3 Bars: A, B above: 100 µm; B below: 50 µm.

Supplementary Table Legends

Table S1. SES *in silico* 3D model.
Sheet 1: I-TASSER output for the top model for each combination as in **Fig. S1A**.
Sheet 2: parameters of the first SES model generated with I-TASSER (**Fig. S1A**).
Sheet 2: parameters of the docking obtained with Haddock for SES model (**Fig. S1B**).

Table S2. RNA-seq in SNB19 cells
Sheet 1: DEGs (both FDR < 0.01 and FC>|1| as in **Fig. 3A** and FDR < 0.1 and FC < 0 as in **Fig. 3F**).
Sheet 2: complete GO analysis (**Fig. 3B**).
Sheet 3: GSEA analysis (hallmark) (**Fig. 3C**).

Table S3. SOX2/SES targets
Sheet 1: SOX2 peaks identified by V5 CUT&Tag
Sheet 2: SES peaks identified by V5 CUT&Tag
Sheet 3: lists of Venn diagram as in **Fig. S4C**.
Sheet 4: lists of genomic regions of **Fig. 3D**-cluster1, used for the heat-map in **Fig. S4F**

Table S4. TFBS enrichment
Sheet 1: Output (statistically significative) of HOMER (Hypergeometric Optimization of Motif EnRichment) analysis on SOX2-V5 CUT&Tag peaks.
Sheet 2: Output (statistically significative) of HOMER (Hypergeometric Optimization of Motif EnRichment) analysis on SES-V5 CUT&Tag peaks.

Table S5. RNA-seq and targets
Sheet 1: lists of Venn diagram as in **Fig. 3F.**
Sheet 2: complete GO analysis (**Fig. 3G**).

Table S6. RNA-seq in U87 cells
Sheet 1: DEGs (both FDR < 0.01 and FC>|1| as in **Fig. S7D**, and FDR < 0.1 and FC < 0 as in **S7G**).
Sheet 2: lists of Venn diagram as in **Fig. S7G**.

Table S7. RNA-seq in neuronal cell cultures
Sheet1: DEGs (FDR < 0.01 and FC>|1|).
Sheet 2: lists of Venn diagram as in **Fig. 6C**.

Table S8. Cell models
Summary of the cell models used in this work and their main features.

Table S9. Antibodies
Details of the antibodies used in this work.

Table S10. Primers

Sequences of primers used in this work.
